# Synthesis of peptide–siRNA conjugates via internal sulfonylphosphoramidate modifications and evaluation of their *in vitro* activity

**DOI:** 10.1093/nar/gkad1015

**Published:** 2023-11-16

**Authors:** Jakob Melgaard Smidt, Lennart Lykke, Carsten Enggaard Stidsen, Nuša Pristovšek, Kurt V Gothelf

**Affiliations:** Interdisciplinary Nanoscience Center (iNANO) and Department of Chemistry, Aarhus University, 8000 Aarhus, Denmark; Research Chemistry, Novo Nordisk A/S, Novo Nordisk Park, 2760 Måløv, Denmark; Centre for Functional Assays and Screening, Novo Nordisk A/S, Novo Nordisk Park, 2760 Måløv, Denmark; Centre for Functional Assays and Screening, Novo Nordisk A/S, Novo Nordisk Park, 2760 Måløv, Denmark; Interdisciplinary Nanoscience Center (iNANO) and Department of Chemistry, Aarhus University, 8000 Aarhus, Denmark

## Abstract

Conjugates of therapeutic oligonucleotides (ONs) including peptide conjugates, provide a potential solution to the major challenge of specific tissue delivery faced by this class of drugs. Conjugations are often positioned terminal at the ONs, although internal placement of other chemical modifications are known to be of critical importance. The introduction of internal conjugation handles in chemically modified ONs require highly specialized and expensive nucleoside phosphoramidites. Here, we present a method for synthesizing a library of peptide–siRNA conjugates by conjugation at internal phosphorous positions via sulfonylphosphoramidate modifications incorporated into the sense strand. The sulfonylphosphoramidate modification offers benefits as it can be directly incorporated into chemically modified ONs by simply changing the oxidation step during synthesis, and furthermore holds the potential to create multifunctionalized therapeutic ONs. We have developed a workflow using a novel pH-controlled amine-to-amine linker that yields peptide–siRNA conjugates linked via amide bonds, and we have synthesized conjugates between GLP1 peptides and a HPRT1 siRNA as a model system. The *in vitro* activity of the conjugates was tested by GLP1R activity and knockdown of the HPRT1 gene. We found that conjugation near the 3′-end is more favorable than certain central internal positions and different internal conjugation strategies were compared.

## Graphical Abstract

**Graphical Abstract:**
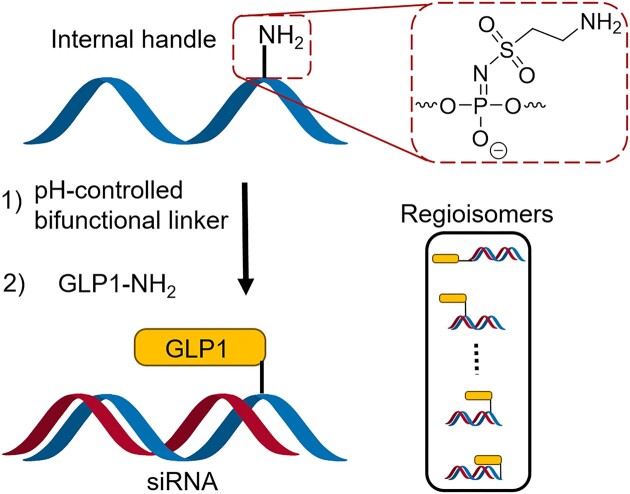

## Introduction

Therapeutic oligonucleotides (ONs) such as antisense ONs (ASOs) and small interfering RNA (siRNA) exhibit remarkable potential in targeting diseases that have been considered intractable by conventional drugs (1,2). One of the major challenges in utilizing ONs as drugs lies in achieving effective delivery of these drugs to the target tissues (3–5). ON-conjugation to targeting ligands holds the potential to solve this challenge (6–9), and a highly successful example of this is the conjugation of *N*-acetylgalactosamine (GalNAc) to ASOs or siRNAs, which allows tissue specific uptake to hepatocytes in the liver (10–12). Other ON conjugates have also showed potential, such as conjugates to fatty acids (13–15), cholesterol (16,17), antibodies (18–20) or peptides (21–23). Specific delivery of ASOs to pancreatic β-cells was recently achieved by conjugation of glucagon like peptide-1 (GLP1) analogues, which showed efficient ligand-induced internalization upon binding to the GLP1 receptor (GLP1R) (24,25). Conjugates of ONs are often linked at the ends of the ON, but it has been suggested that peptide conjugation through internal positions can improve target recognition and increase the stability towards enzymatic degradation (26,27). It is also well- known that the positioning of chemical modifications is extremely important and can drastically change the properties of therapeutic ONs (28–31). Albeit, conjugation is most often performed at the termini of the ON, since internal conjugation handles can be cumbersome to synthesize due to the requirement for special phosphoramidites for each different nucleotide. This is in particular the case for chemically modified nucleotides used in therapeutic ONs. Conjugation at the terminal positions of the ON also naturally limits the number of conjugation sites and prohibits multifunctional conjugates with several different ligands. We recently reported a method for incorporating internal chemical handles into DNA ONs using sulfonyl azides (32), and this presents an opportunity to simplify the synthesis of therapeutic ONs with internal conjugation sites. Here, we present a workflow for synthesizing a library of regioisomeric peptide–ON conjugates, where the ONs carry amino-groups linked via sulfonylphosphoramidates at internal positions. We also present a pH-controlled amine-to-amine linker, which enables easy conjugation of the amino-modified ONs to the lysine in a peptide. In this study, we use a fully chemically modified siRNA against the hypoxanthine phosphoribosyltransferase 1 gene (HPRT1) and conjugate the siRNA to GLP1 analogues as a model system for our workflow. These methodologies can be readily expanded to encompass other peptide–ON conjugates, and to incorporate a wide array of other internal modifications into therapeutic ONs.

## Materials and methods

### Solid phase oligonucleotide synthesis

Sense strands were synthesized on a Bioautomation MerMade 192X synthesizer using 1000 Å 1 μmol universal support CPG columns with standard coupling conditions. Phosphoramidites were purchased from Hongene Biotech, except phosphoramidites used for 2′ and C5 amino linker incorporation which were purchased from LGC Biosearch Technologies. Antisense strands and 3′-modified strands were synthesized on an ÄKTA Oligopilot Plus 10 synthesizer using either Glen UnySupport™ PS or 3′-amino-modifier CPG support. Sulfonylphosphoramidate modifications were made by replacing the regular oxidation step for treatment with a 0.45 M solution of compound **1** in acetonitrile or 1:1 acetonitrile/pyridine. Deprotection and cleavage from the solid support was carried out using AMA (1:1 32% ammonium hydroxide/40% methylamine). ONs synthesized on the Oligopilot were purified by HPLC (method A). ONs synthesized on the MerMade were purified using standard DMT-on procedure with GlenPak DNA purification cartridges. ONs were then lyophilized before storage in freezer.

### Yield determination of sulfonylphosphoramidate modifications

ON 8-mers made of either DNA, 2′OMe-modified, or 2′F-modified nucleotides were synthesized on the MerMade system. The final nucleoside was modified by switching the regular oxidation step for treatment with a 0.45 M solution of compound **1** in acetonitrile. Treatment times were either 2 × 5 min, 2 × 15 min, or 4 × 15 min. After cleavage and deprotection as described above, the ONs were analyzed by HPLC (method B). The reaction yield of the sulfonyl azide reaction was determined from the integrals of the two peaks corresponding to the full-length strand with and without sulfonylphosphoramidate modification. Any truncated strands were ignored in this regard.

### Solid phase peptide synthesis

Peptides were synthesized on a SymphonyX peptide synthesizer at 450 μmol scale from either a Fmoc-Lys(Boc)-Wang resin or a Fmoc-Gly-Wang resin using 3 h coupling steps with *N*,*N*’-diisopropylcarbodiimide and Oxyma Pure and deprotection with 20% piperidine in DMF. After synthesis, the peptides were treated with a cleavage mixture (95% TFA, 2% H_2_O, 2% TIPS, 1 w/v% DTT) for 2 h, followed by precipitation and washing in Et_2_O. Peptides were then dissolved in 1:1 AcOH/H_2_O and purified by HPLC (Method C) before lyophilization.

### Peptide-siRNA conjugation

Three different methods were utilized to fabricate GLP1–siRNA conjugates. All compounds tested further were produced by method 3.

Method 1: ‘One pot procedure’. 500 nmol ON was dissolved in 75 μl tetraborate buffer (50 mM, pH 8.5) and 2 eq. of compound **2** was added (4 mg/100 μl dissolved in DMSO) and the reaction was incubated for 40 min at 25°C. Then, 4 eq. peptide was added (2 μmol/300 μl dissolved in 0.2 M phosphate buffer pH 11.3) and the reaction was incubated for 3 h at 25°C followed by neutralization by adding 50 μl 2 M TEAA buffer pH 7. The conjugate was then purified by HPLC (method D) (34 to 43% overall yield).

Method 2: ‘Spinfilter procedure’. 500 nmol ON was dissolved in 75 μl tetraborate buffer (50 mM, pH 8.5) and 4 eq. of compound **2** was added (4 mg/100 μl dissolved in DMSO) and the reaction was incubated for 40 min at 25°C. The reaction mixture was purified with an Amicon 3K spinfilter using PBS. Then, 2 eq. peptide was added (1 μmol/250 μl dissolved in 0.2 M phosphate buffer pH 11.3) and the reaction was incubated for 3 h at 25°C followed by neutralization by adding 50 μl 2 M TEAA buffer pH 7. The conjugate was then purified by HPLC (method D) (36 to 50% overall yield).

Method 3: ‘HPLC procedure’. 500 nmol ON was dissolved in 75 μl tetraborate buffer (50 mM, pH 8.5) and 4 eq. of compound **2** was added (4 mg/100 μl dissolved in DMSO) and the reaction was incubated for 40 min at 25°C. The DCSP-modified ON was purified by HPLC (method E) and relevant fractions were lyophilized. Then, the ON was dissolved in 90 μl PBS and 2 eq. peptide was added (1 μmol/250 μl dissolved in 0.2 M phosphate buffer pH 11.3) and the reaction was incubated for 3 h at 25°C followed by neutralization by adding 50 μl 2 M TEAA buffer pH 7. The conjugate was then purified by HPLC (method D) (8 to 29% overall yield).

To generate the final GLP1–siRNA conjugates, the peptide-modified sense strands were mixed 1:1 molar ratio with the corresponding antisense strand in PBS.

### GLP1R activity assay

GLP1R activity was measured through cyclic adenosine monophosphate (cAMP) potency using a clonal BHK cell line that stably co-express human GLP1R and the cAMP response element (CRE) luciferase reporter gene, essentially as previously reported (33). Briefly, frozen stocks of assay-ready cells were thawed to 37°C, washed once with PBS, and diluted to 100 000 cells/ml in assay buffer (DMEM without phenol red supplemented with 1× GlutaMAX, 10 mM HEPES, 1 w/v% ovalbumin, and 0.1 v/v% Pluronic F-68). Serial dilutions (10-fold dilutions in assay buffer, 8 concentrations for each compound) were made for GLP1–siRNA conjugates and free peptides in 96 well dilution plates using a Biomek i7 liquid handler. Then, 50 μl of each dilution were transferred to 96-well assay plates to which 50 μl of the diluted cell suspension was added (5000 cells/well). Plates were incubated for 3 h at 37°C in 5% CO_2_, then left at room temperature for 5 min before 100 μl SteadyLite Plus (PerkinElmer) was added to each well. Plates were sealed from light and incubated at room temperature for 30 min while gently shaking before luminescence was detected on a Biotek Synergy 2 plate reader. Half maximal effective concentration (EC50) and maximal effect (*E*_max_) values were calculated from non-linear curve fitting using a four-parameter logistic function (Hill slope = 1) using TIBCO Enterprise Runtime for R. *E*_max_ values were normalized to percentage of an internal GLP1 control.

### HPRT1 knockdown studies

HeLa cells (20 000 cells/well) were seeded for 24 h in a PDL-coated 96 well plate. GLP1–siRNA constructs or free siRNA were diluted in optiMEM to the desired concentration, and cells were transfected using Lipofectamine RNAiMAX according to manufacturer's protocol. Cells were incubated for 48 h (for Figures 4C and 5B) or 24 h (for Figure 4D). RT-qPCR analysis was carried out using the Fast Advanced Cells-to-CT kit (Invitrogen) following manufacturer's protocol. Briefly, cells were washed with 50 μl PBS and then lysed with 50 μl lysis buffer for 5 min at room temperature before adding 5 μl stop solution. Then, 40 μl RT reaction mix was mixed with 10 μl cell lysate and reverse transcription was performed to produce cDNA. The cDNA was diluted 2-fold in nuclease free water and 4.5 μl diluted cDNA was mixed with 5.5 μl qPCR cocktail master mix (4.5 μl Taqman Fast Advanced Master Mix, 0.5 μl HPRT-FAM Taqman Gene Expression assay Hs02800695_m1, 0.5 μl UBC-VIC Taqman Gene Expression assay Hs05002522_g1) in a 384-plate on a Biomek i5 liquid handler. For each well, technical duplicates were made. Samples were then analyzed by RT-qPCR with a QuantStudio 7 Pro Real-Time PCR System running in duplex mode with ubiquitin C (UBC) as endogenous control using the ΔΔCt method. Data were normalized to an untreated control and, when relevant, fitted to a four-parameter logistic function from which half maximal inhibitory concentration (IC50) values were acquired.

### Knockdown studies without transfection

MIN6 cells (10 000 cells/well) were seeded for 24 h in a CellBIND 96 well plate (Corning) with growth media (DMEM supplemented with 1× GlutaMAX, 10% FBS and 50 μM β-mercaptoethanol). GLP1–siRNA constructs or free siRNA were added to a final concentration of 1 μM, and a positive control was made by transfection of HPRT1 siRNA with lipofectamine RNAiMAX according to manufacturer's protocol. After incubation for 48 h, RT-qPCR analysis was carried out as described above, except using murine Taqman probes (HPRT-FAM Mm03024075_m1 and UBC-VIC Mm02525934_g1).

### Melting studies

GLP1–siRNA conjugates and free siRNA were diluted to 1 μM in buffer (10 mM sodium phosphate pH 7, 100 mM sodium chloride). The siRNA duplex melting was followed by 260 nm UV on a Themo Scientific Evolution 260 Bio spectrophotometer while heating from 30°C to 95°C (1°C pr. min) with a Thermo Scientific PCCU1 temperature controller. Melting curves were normalized and the first derivative was calculated and smoothened using LOWESS smoothening (medium, 10 points in smoothening window) in GraphPad Prism 9. Melting temperatures (*T*_m_) were found at the maxima of the smooth first derivative and difference in T_m_ compared to free HPRT1 siRNA (Δ*T*_m_) was calculated.

## Results and discussion

### Synthesis of a phthalimide-containing sulfonyl azide for generating internal amino-modifications

**Figure 1.**
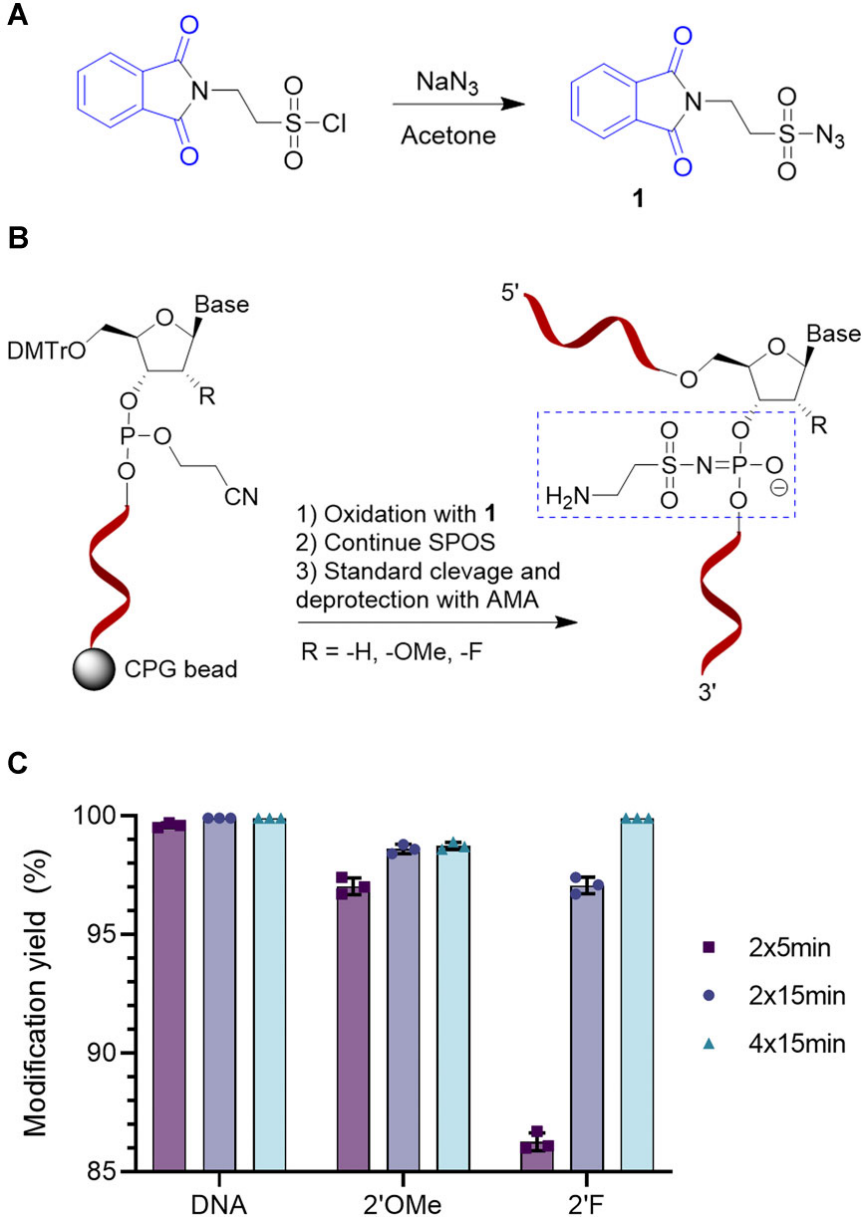
Synthesis and reactivity of compound **1**. (**A**) Synthesis of **1** is performed in a single step from 2-phthalimidoethanesulfonyl chloride. (**B**) Internal amino-modification of ONs is done by replacing the regular oxidation step for treatment with **1** during SPOS. (**C**) Modification yield was evaluated by HPLC analysis after SPOS of a DNA, 2′OMe or 2′F modified 8-mer ON with a single amino-modification made by treatment with **1**. Data represents mean and standard deviation (SD) of *n* = 3 replicated syntheses.

We recently reported on multiple different sulfonyl azides for incorporation of different chemical handles, including amines, internally into DNA ONs (32). These amines were protected by either trifluoroacetyl (TFA) or fluorenylmethoxycarbonyl (Fmoc) and the resulting reagents were both useful for labeling, but they were also associated with some challenges. Especially better solubility, faster reaction time, and a scalable purification procedure would be desirable when designing a new reagent. We therefore synthesized the new sulfonyl azide **1** containing a phthalimide which encompasses the desired properties (Figure 1A). The phthalimide serves as a protecting group during solid phase ON synthesis (SPOS) and affords an amine upon standard deprotection and cleavage with ammonia or methylamine (Figure 1B). Compound **1** is synthesized in one step from 2-phthalimidoethanesulfonyl chloride by reaction with NaN_3_. It is possible to recrystallize **1** from ethanol which allows for simple large-scale purification, and we successfully recrystallized multiple batches on 20–30 g scales.

Modification of ONs with **1** was tested at different reaction times during the oxidation step, followed by analysis of the deprotected ONs by HPLC to assess the modification yield. DNA ONs were >99% modified after treatment with **1** for 2 × 5 min, but for 2′OMe and 2′F modified ONs, longer oxidation times were required to obtain high conversion (Figure 1C). Similarly, the requirement for longer oxidation times have also been reported for oxidizing sterically hindered analogues with mesyl azide (31). Notably, the resulting sulfonylphosphoramidate generates a new stereocenter at phosphorus unlike the regular phosphodiester backbone, and therefore the produced ON is a mixture of diastereomers, similar to that of ONs modified with phosphorothioates (34).

### Preparation of GLP1–siRNA conjugates by a pH-controlled amine-to-amine linker

**Figure 2.**
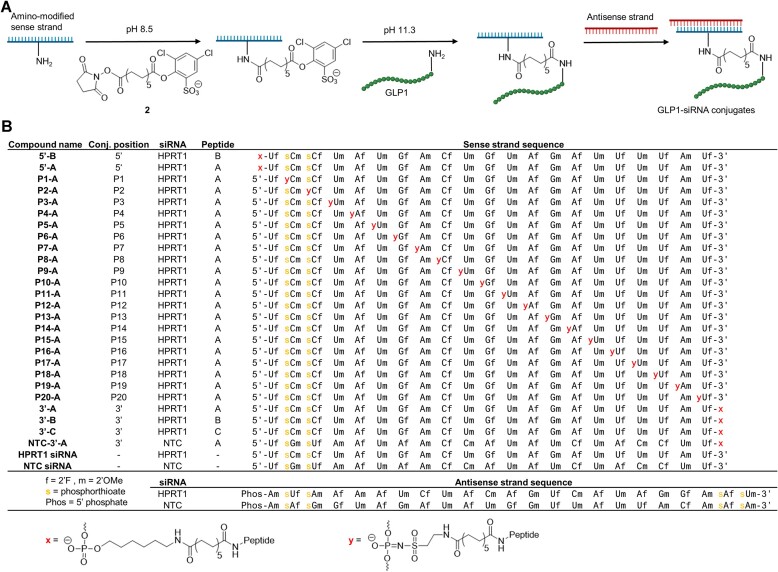
Regioisomeric GLP1–siRNA conjugates. (**A**) Workflow for generating conjugates. Sense strands were amino-modified at different positions in the backbone using **1** during SPOS. The pH-controlled linker **2** was used to conjugate the sense strands to GLP1 followed by hybridization with the corresponding antisense strand to form GLP1–siRNA conjugates. (**B**) List of synthesized strands and chemical structure of modification.

Bifunctional linkers employed in bioconjugation typically harness the orthogonal reactivity of distinct functional groups in biomolecules. For instance, primary amines have the propensity to react with activated esters, while thiols exhibit a marked reactivity towards electrophilic entities, including maleimides. This capacity for selective reactivity provides a strategic pathway for conjugation (35). However, some reaction products have unfavorable intrinsic properties when used for drug delivery such as sulfides that are prone to thiol exchange by retro-Michael addition (36). The reaction between amines and activated esters affords amide bonds which are naturally found in peptides and provides stable conjugates. For this purpose, we designed and synthesized a new pH-controllable amine-to-amine linker (compound **2**) that yields peptide–ON conjugates joined by two amide bonds (Figure 2A, Supplementary Figure S1A). Compound **2** consists of an *N*-hydroxysuccinimide (NHS) ester, a C12 spacer, and an activated ester based on 2,4-dichloro-6-sulfonic acid phenol (DCSP), which was recently used for selective modification and cross-linking of peptides (37,38).

Amino-modified ONs are able to react with the NHS ester at pH 8.5 without reacting with the DCSP ester (Supplementary Figure S1B). Subsequently, the pH can be increased to 11.3, at which the DCSP ester rapidly reacts with the ϵ-amino group of lysine in a peptide. This linker can therefore join two amine-containing reaction partners in a pH-controlled manner.

**Figure 3.**
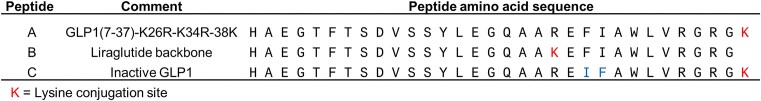
Sequences of GLP1 analogues.

With SPOS, we synthesized a library of amino-modified HPRT1 siRNA sense strands (19,39) that were either modified at the terminal positions using standard linkers (linkage **x**) or at different internal positions throughout the backbone by using **1** to provide linkage **y** (Figure 2B). The ONs were then converted to the DCSP-carrying derivatives by reaction with **2**, followed by conjugation to different GLP1 analogues to form peptide–ON conjugates. Three different peptides were used: A GLP1 analogue carrying a C-terminal lysine and in which the other lysines were mutated to arginine (peptide A, Figure 3), an analogue based on the Liraglutide peptide backbone with a single internal lysine (peptide B), and an inactive GLP1 similar to peptide A but with a key phenylalanine swapped with the neighboring isoleucine to inactivate binding to the GLP1R (peptide C) (40–42).

The crude DCSP-carrying ONs can be directly conjugated to GLP1 in a one-pot manner by using an excess of the peptide. Alternatively, the DCSP-carrying ONs can be purified by simple ultrafiltration filters or by HPLC and then subsequently reacted with the peptide to form GLP1–siRNA conjugates (see Materials and Methods). The DCSP-carrying ONs were stable for several months in the freezer after purification. Following peptide conjugation, the ON–peptide conjugates were purified by HPLC and hybridized with the corresponding antisense strand to form the final GLP1–siRNA conjugates (Figure 2B). A conjugate using a non-target control (NTC) siRNA was also made.

### GLP1R activity and gene knockdown with GLP1–siRNA conjugates

**Figure 4.**
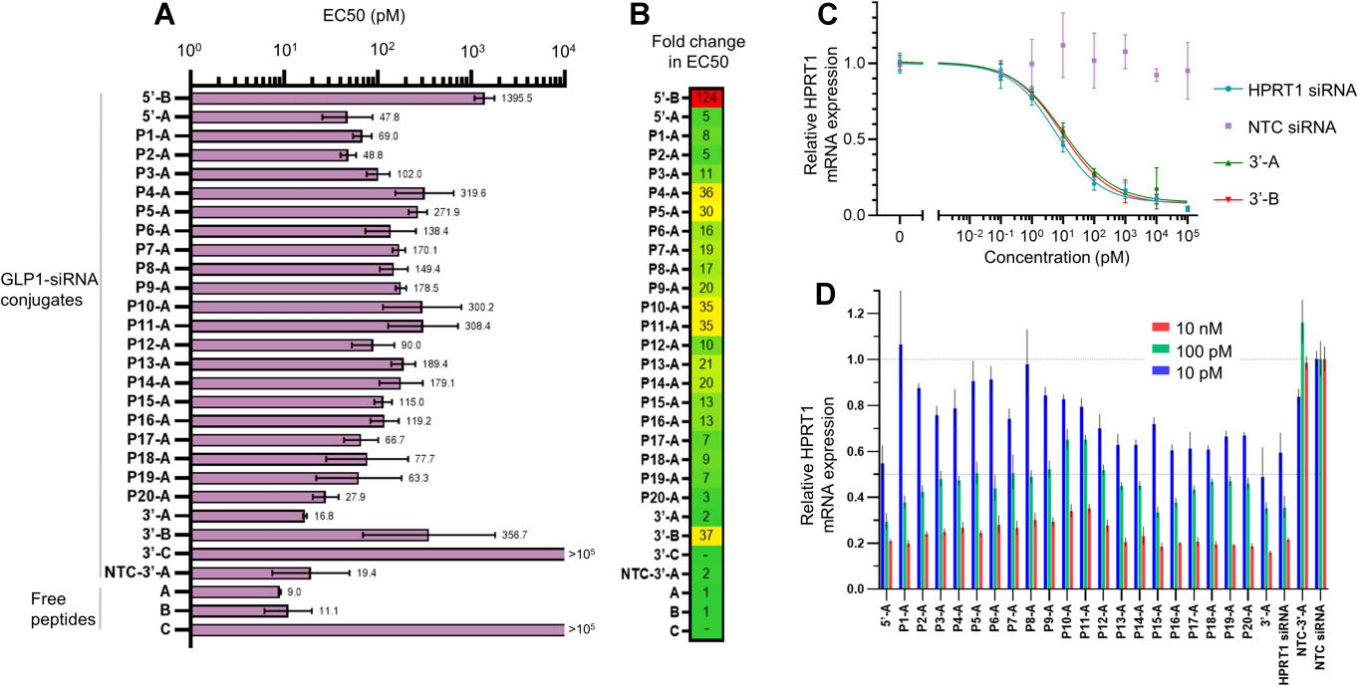
In vitro activity of regioisomeric GLP1–siRNA conjugates. (**A**) Activation of GLP1R was measured with a CRE/Luciferase reporter assay in BHK cells expressing human GLP1R. EC50 values are presented as mean and 95% CI of n = 3 independent experiments. (**B**) Fold change in EC50 compared to free peptide. (**C**) Gene knockdown dose-response with GLP1–siRNA conjugates measured by RT-qPCR. Data points represents mean and SD of *n* = 5 biological replicates. (**D**) Gene knockdown with internally modified GLP1–siRNA library at three different concentrations (10 pM, 100 pM and 10 nM) measured by RT-qPCR. Data presented as mean and SD of *n* = 3 biological replicates.

We next investigated how the regiospecific conjugation of siRNA to GLP1 interferes with the peptides ability to bind and activate GLP1R. This is performed by using a luciferase reporter under the control of CRE in recombinant BHK cells constitutively expressing human GLP1R. Binding of GLP1 to GLP1R results in elevated intracellular cAMP levels which activates the CRE binding protein (CREB) in turn leading to binding of CRE and expression of reporter luciferase. Dose response curves for all GLP1–siRNA conjugates were measured and fitted to four-parameter logistic functions from which EC50 and *E*_max_ values were calculated (Figure 4A, Supplementary Figure S4). The fold changes in EC50 from the parent free peptides were also calculated (Figure 4B). All conjugates showed reduction in potency although only slight attenuation was observed for conjugation at or near the 3′ and 5′ terminal positions. Conjugation in the center positions still showed potent EC50 values in the ∼100–300 pM range.

For the conjugation position in the peptide, it was interestingly observed that conjugation of siRNA through the internal lysine of peptide B significantly reduced potency. This could be due to unfavorable interactions at the peptide N-terminal, which is crucial for GLP1R binding (43), as the siRNA is closer to the N-terminal in peptide B. Increasing linker length in these conjugates could potentially allow for less disruption (33).

To investigate whether internal conjugation of a peptide to the siRNA interfered with the ability to cause gene knockdown, we performed transfection of GLP1–siRNA conjugates in HeLa cells followed by RT-qPCR analysis of the HPRT1 mRNA. Dose-response curves were made for the conjugates carrying peptide A or B at the 3′ position (Figure 4C), and we observed that peptide conjugation at this position did not interfere with knockdown of the target gene when compared to the free HPRT1 siRNA.

Knockdown by the library of internally modified GLP1–siRNA conjugates were then tested at three different concentrations (Figure 4D). Conjugation was generally well tolerated at all positions, although some positions, especially in the middle (P10-A, P11-A), showed less knockdown even at high concentrations. Conjugation at the terminal positions of the RNA sequence was generally accepted, and it appears that especially constructs with conjugation positions near the 3′-end are largely similar in their ability to cause gene knockdown compared to free HPRT1 siRNA.

We also attempted knockdown without a transfection agent in the murine pancreatic β-cell line MIN6, by treating cells with a high concentration (1 μM) of the regioisomeric library of GLP1–siRNA conjugates. However, we did not observe significant levels of knockdown for any of the constructs compared to the NTC conjugate (Supplementary Figure S2). This suggests that receptor-mediated productive uptake of GLP1–siRNA conjugates is not as efficient as for GLP1-ASO conjugates observed by others (24). This is likely due to poor endosomal escape of the siRNA, whereas ASOs are known to enter the cell cytoplasm through direct gymnotic uptake (44,45). Effective endosomal escape of siRNA is a challenge that has to be addressed if this approach has to be used therapeutically.

### Comparison to other internal conjugation strategies

**Figure 5.**
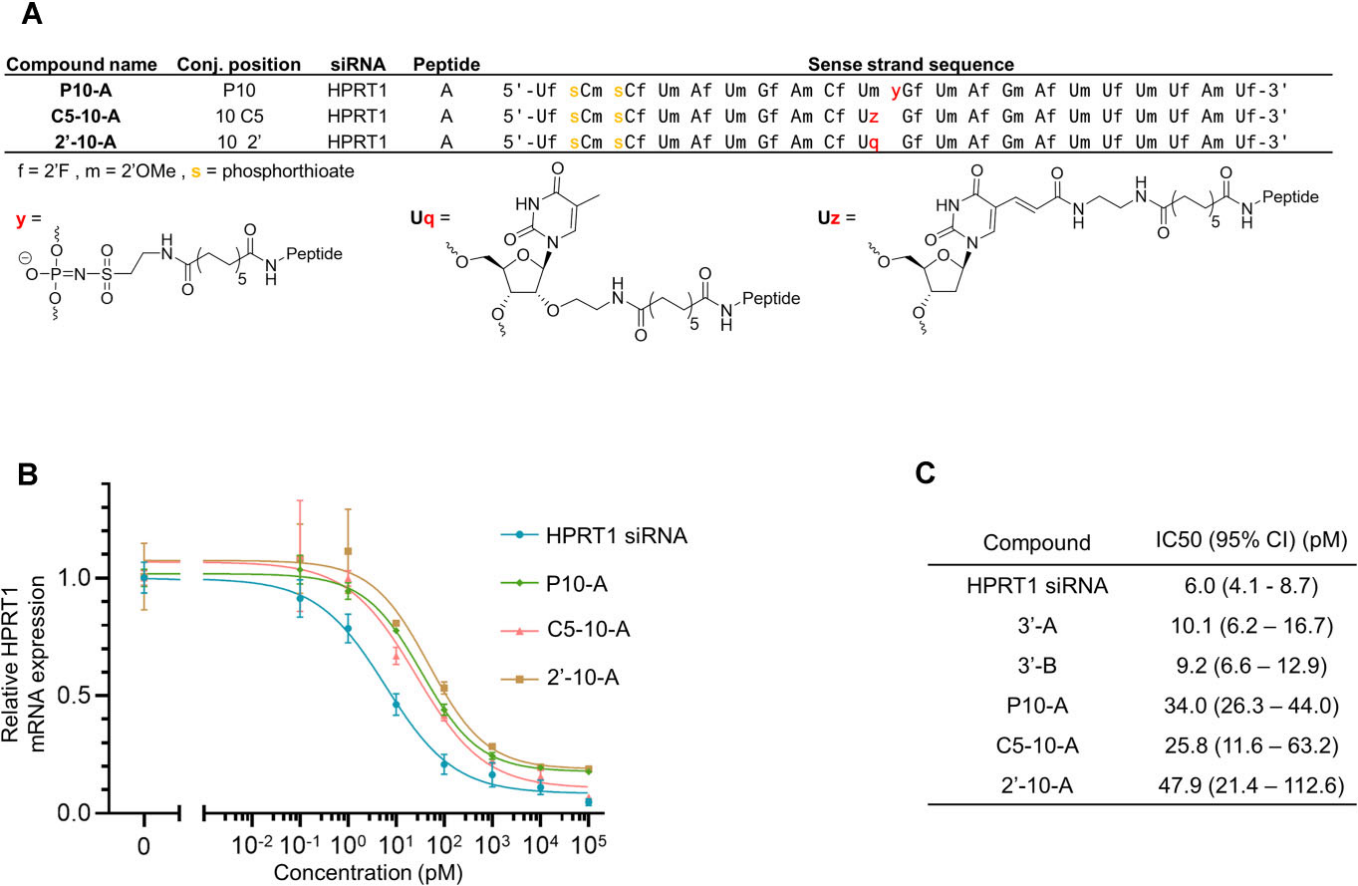
Comparison between different internal modification strategies. (**A**) Structure of internal modifications at position 10 of GLP1–siRNA conjugates. (**B**) Gene knockdown dose-response curves measured with RT-qPCR. Data points represents mean and SD of *n* = 3 or *n* = 5 (HPRT1 siRNA) biological replicates. (**C**) IC50 values for gene knockdown from dose–response curves.

In a recently reported study, it was shown that internal 2′ conjugation of a C16 lipid in certain central positions (positions 9, 10 and 11) of the siRNA sense strand caused large disturbances in the ability to cause gene knockdown even at high concentrations (46). In our current study, we utilized a similar siRNA design strategy, which resulted in successful knockdown for all conjugates featuring internal sulfonylphosphoramidate modifications. This finding subsequently prompted us to investigate how other internal modification methods might compare with our established GLP1–siRNA conjugates. Two new GLP1–siRNA conjugates were synthesized, where the peptide was linked through either the 2′ position or C5 in the nucleobase at the internal position 10 (Figure 5A).

These were compared to P10-A, which was the least active of the sulfonylphosphoramidate modified GLP1–siRNA conjugates. Dose response curves of gene knockdown was made for all three conjugates (Figure 5B) and IC50 values were found for all dose response curves (Figure 5C). Interestingly, we observed a similar dose response for the three internal modifications and did not observe the anticipated low degree of knockdown for the 2′ modified conjugate at this position.

The ability of the modified siRNA to cause knockdown may therefore be more dependent on either sequence or type of modification instead of the internal conjugation position. The internal sulfonylphosphoramidate modification does, however, offer benefits over the other types of internal modifications, because its incorporation in modified ONs is highly feasible. The chemical handles can be incorporated directly into ONs synthesized with modified phosphoramidites by simply changing the oxidation step during SPOS, whereas for chemical handles at the 2′-position and nucleobase, a completely new phosphoramidite must be synthesized if a modified version is desired. The synthesis of sulfonyl azides requires only one or few steps, while modified phosphoramidites with chemical handles are often not commercially available and can involve multistep synthesis routes such as for 2′-handles (47), locked nucleic acid (LNA) (48) or 2′-OMe phosphoramidites with conjugation handles (49).

### Melting study of siRNA duplex in GLP1–siRNA conjugates

Melting temperatures (*T*_m_) of the siRNA duplexes of all GLP1–siRNA conjugates were measured to investigate the effect of peptide conjugation on the duplex stability (Supplementary Figure S3A). It was observed that the peptide conjugation caused slight destabilization of the siRNA duplex by a decrease in the melting temperature of up to 4°C compared to free HPRT1 siRNA (Δ*T*_m_). The effect is increased when the peptide is conjugated in the middle positions (Supplementary Figure S3B). Conjugation near the 3′-end only cause little to no effect on the melting temperature. Melting temperatures of the conjugates made with different conjugation strategies at position 10 revealed that conjugation through the sullfonylphosphoramidate and the C5 position in the nucleobase caused a larger destabilization than peptide conjugation through the 2′ position (Supplementary Figure S3C). This tendency to destabilization could become problematic if the effect is additive and multiple modifications are incorporated. However, all GLP1–siRNA conjugates had acceptable melting temperatures of higher than 76°C (Supplementary Figure S4).

## Discussion

The positioning of chemical modifications in therapeutic ONs has a major impact on their therapeutic properties. Similarly, the positioning of the conjugation site in ON conjugates could potentially be important. The workflow presented here allows introduction of internal modifications throughout the backbone of chemically modified ONs in a simple and feasible manner without the need to synthesize specialized phosphoramidite reagents. The method is compatible with well plate formats, so the method can easily be employed in screening efforts. We used HPRT1 siRNA and a GLP1 peptide as a model system, but the method could in principle be used for conjugation of other peptides, ligands, or fatty acids, and thereby be useful for studying the difference between regioisomeric conjugates. Multiple different ligands or functionalities could also be combined to generate multifunctional therapeutic ON conjugates, and we have previously shown that different orthogonal chemical handles can be incorporated in the same strand to allow for multifunctionalization of DNA (32). Potentially, ligands that enhance tissue targeting, pharmacokinetics, and endosomal escape could be combined on the ON using our presented conjugation methodology.

The presented pH-controllable active ester combination showed excellent performance in creating peptide–ON conjugates linked via amides. Yet, the high pH used for conjugation of the peptide could prove problematic if used with unmodified RNA, as RNA is unstable at elevated pH. However, therapeutic ONs are often chemically modified, and we did not observe issues with stability of our ONs under these conditions (Supplementary Figure S5). Alternatively, conjugation of unmodified RNA could potentially be performed before removing the 2′-protecting group which is necessary during SPOS. In addition, the employed GLP1 peptides have excellent chemical stability at elevated pH (Supplementary Figure S6), however deamidations or other degradation pathways should be considered applying this methodology on other peptides.

Agonist mediated endocytosis of the GLP1R has been widely studied, in the context of understanding the link between receptor activation, signaling pathway, and receptor recycling kinetics in relation to insulin secretion (50). However, in the context of employing the GLP1R as a transporter to the cytosol literature is scarce. Interestingly, Fang *et al.* has described the influence of different GLP1R agonists on receptor trafficking, showing pronounced differences in receptor recycling or degradation depending on the agonist (51). For instance, exendin-4 is recycled much slower than GLP1, while mutations near the N-terminal of the GLP1R agonists also impact recycling kinetics significantly. To enable successful cytosolic delivery of GLP1–siRNA conjugates, this hints towards searching for optimized GLP1 analogues with desired properties regarding endosomal release. Understanding GLP1R trafficking is an important aspect of pancreatic siRNA targeting, however the endosomal escape remains to be a challenge. Peptide–ON conjugates for cytosolic delivery, utilizing cell penetrating peptides have been widely studied, however it is debated whether this is a universally applicable strategy as molecules that increase the leakiness of endosomes have a narrow therapeutic index because permeabilizing endosomes and lysosomes is toxic. It has recently been illustrated that ‘halting’ endosomes at a pre-lysosomal stage where membrane fission and fusion occur at a high rate increase ON activity significantly (52). It is believed that vesicle budding and fusion is an important site for ON escape, as these dynamic membrane remodeling events, create non-bilayer regions that have increased permeability.

Therapeutic ONs hold the potential to revolutionize medicine by unlocking the intracellular target space, however extrahepatic tissue specific targeting remains a challenge. Here we have demonstrated new ON-peptide conjugation chemistry enabling future work exploring the therapeutic value of tissue targeted siRNA.

## Supplementary Material

gkad1015_Supplemental_File

## Data Availability

The data underlying this article are available in the article and in its online supplementary material. Further raw data underlying this article will be shared on reasonable request to the corresponding author.
