# Supplementary material for: Synthesis of peptide–siRNA conjugates via internal sulfonylphosphoramidate modifications and evaluation of their in vitro activity
Source: Nucleic Acids Res. 2023 Nov 16;52(1):49–58. doi: 10.1093/nar/gkad1015 (PMC10783514; doi:10.1093/nar/gkad1015)
Supplement: gkad1015_Supplemental_File [file gkad1015_supplemental_file.pdf]

Supplementary Information

Synthesis of peptide-siRNA conjugates via internal sulfonylphosphoramidate modifications and evaluation of their *in vitro* activity

Jakob Melgaard Smidt[1], Lennart Lykke[2]*, Carsten Enggaard Stidsen[3], Nuša Pristovšek[3], Kurt V. Gothelf[1]*.

[1] Interdisciplinary Nanoscience Center (iNANO) and Department of Chemistry, Aarhus University, 8000 Aarhus, Denmark

[2] Research Chemistry, Novo Nordisk A/S, Novo Nordisk Park, 2760 Måløv, Denmark.

[3] Centre for Functional Assays and Screening, Novo Nordisk A/S, Novo Nordisk Park, 2760 Måløv, Denmark.

Supplementary figures

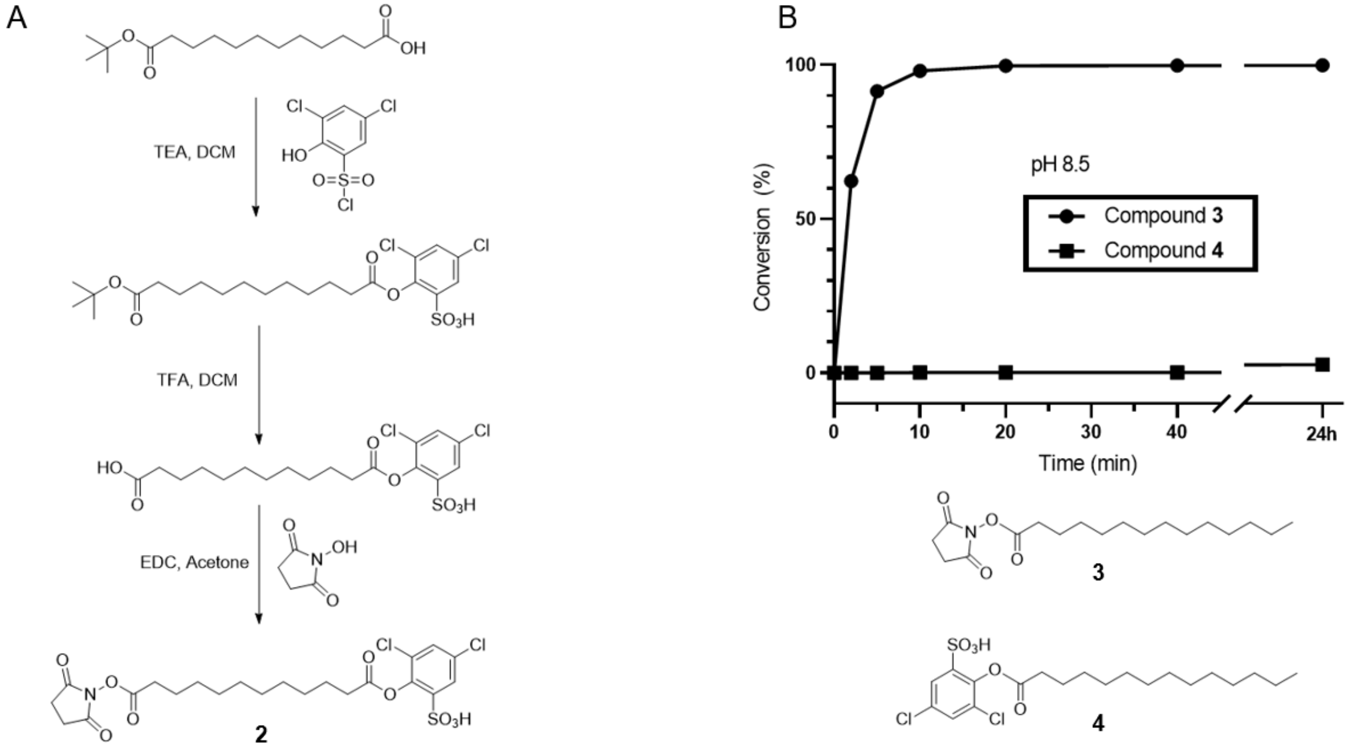

Supplementary Figure S1. Synthesis of compound **2** and reactivity of NHS and DCSP linkers. (**A**) Synthesis of compound **2**. (**B**) Reaction conversion of NHS-C14 (compound **3**) and DCSP-C14 (compound **4**) linker reacting with amine-modified oligonucleotide at pH 8.5. Conversion determined by HPLC analysis.

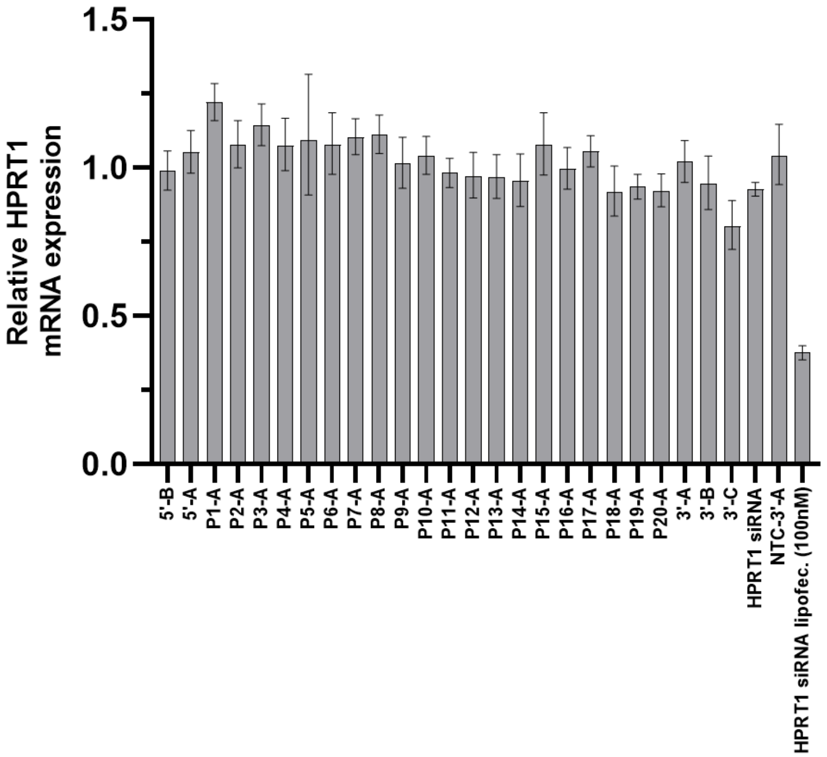

Supplementary Figure S2. Knockdown study without transfection in murine pancreatic β-cells. MIN6 cells were treated with 1 µM of different GLP1-siRNA constructs without the use of a transfection agent. A positive control was made with 100 nM free HPRT1 siRNA using transfection. No productive free uptake was observed for any of the compounds compared to the NTC construct. Data presented as mean and SD of n=3 biological replicates.

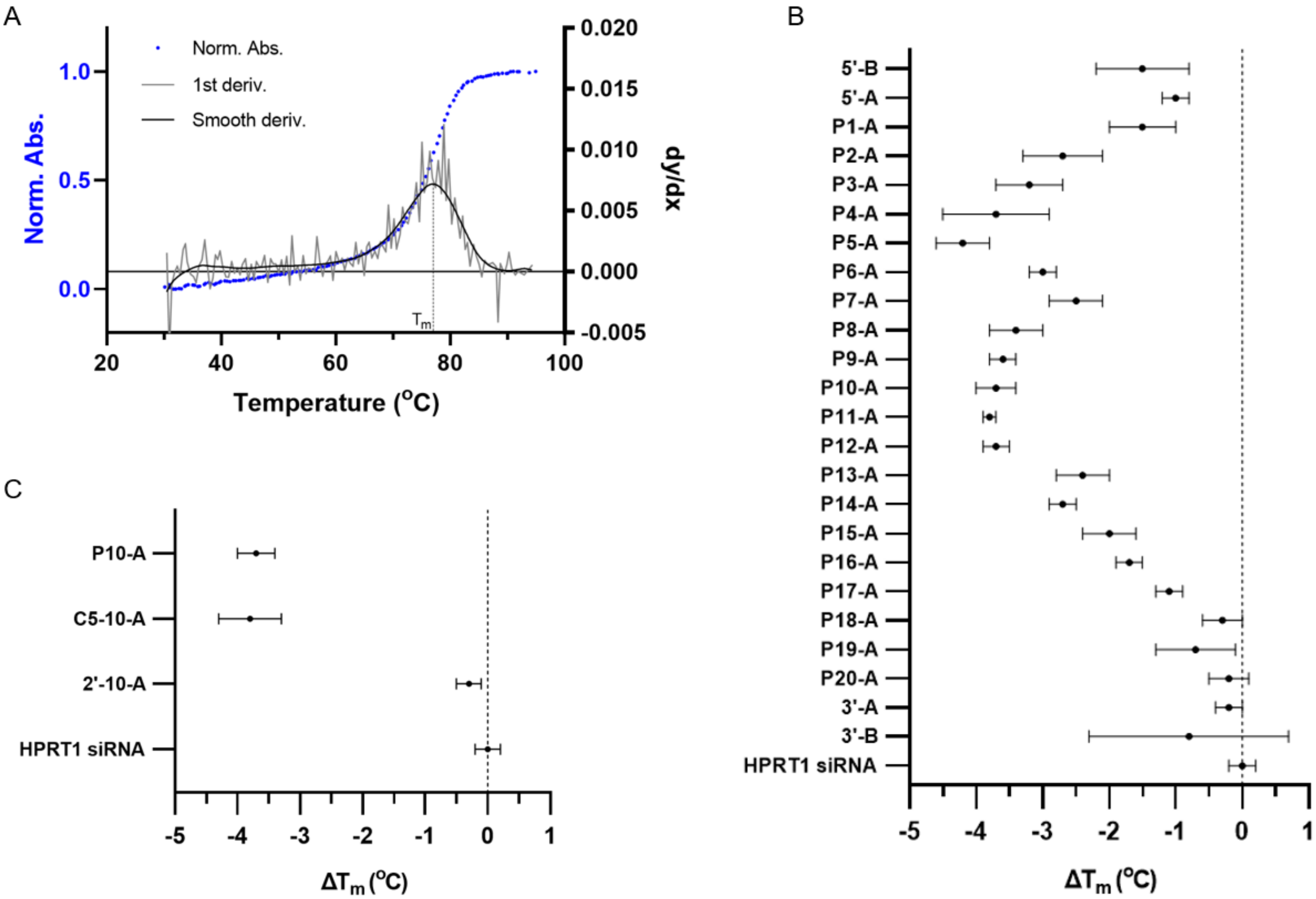

Supplementary Figure S3. Melting study of siRNA duplex in GLP1-siRNA conjugates. (**A**) Representative example of a melting curve of a GLP1-siRNA conjugate. Absorbance was measured at 260 nm and the first derivative was calculated and smoothened. The highest point of the smooth derivative curve was used to determine T_m. (**B**) ΔT_m of regioisomeric GLP1-siRNA conjugates compared to free HPRT1 siRNA. (**C**) ΔT_m of GLP1-siRNA conjugates made with different conjugation strategies at position 10. Data points represent mean and SD from n=3 independent experiments.

Compound name	GLP1R activation EC50 (95% CI) [pM]	Fold change in EC50	E_{max} (±SD) [%]	Melting T_m (±SD) [°C]	ΔT_m (±SD) [°C]	HPRT1 knockdown IC50 (95% CI) [pM]
**5'-B**	1395.5 (1094.8 – 1778.8)	124	100 (± 2)	79.1 (0.7)	-1.5 (0.7)	-
**5'-A**	47.8 (25.8 – 88.6)	5	91 (± 4)	79.6 (0.1)	-1.0 (0.2)	-
**P1-A**	69.0 (54.9 – 86.8)	8	97 (± 8)	79.1 (0.5)	-1.5 (0.5)	-
**P2-A**	48.8 (40.2 – 59.1)	5	98 (± 8)	77.9 (0.6)	-2.7 (0.6)	-
**P3-A**	102.0 (76.9 – 135.3)	11	95 (± 5)	77.4 (0.5)	-3.2 (0.5)	-
**P4-A**	319.6 (156.0 – 654.7)	36	92 (± 6)	76.9 (0.8)	-3.7 (0.8)	-
**P5-A**	271.9 (216.9 – 340.9)	30	91 (± 5)	76.4 (0.4)	-4.2 (0.4)	-
**P6-A**	138.4 (74.4 – 257.5)	16	90 (± 7)	77.6 (0.1)	-3.0 (0.2)	-
**P7-A**	170.1 (145.6 – 198.8)	19	94 (± 8)	78.1 (0.4)	-2.5 (0.4)	-
**P8-A**	149.4 (105.6 – 211.3)	17	94 (± 4)	77.2 (0.4)	-3.4 (0.4)	-
**P9-A**	178.5 (156.3 – 203.9)	20	90 (± 6)	77.0 (0.2)	-3.6 (0.2)	-
**P10-A**	300.2 (114.0 – 790.2)	35	95 (± 4)	76.9 (0.3)	-3.7 (0.3)	34.0 (26.3 – 44.0)
**P11-A**	308.4 (130.5 – 728.2)	35	94 (± 3)	76.8 (0.1)	-3.8 (0.1)	-
**P12-A**	90.0 (53.2 – 152.2)	10	99 (± 3)	76.9 (0.2)	-3.7 (0.2)	-
**P13-A**	189.4 (140.8 – 254.9)	21	97 (± 3)	78.2 (0.3)	-2.4 (0.4)	-
**P14-A**	179.1 (105.1 – 305.5)	20	94 (± 5)	77.9 (0.2)	-2.7 (0.2)	-
**P15-A**	115.0 (92.7 – 142.7)	13	92 (± 6)	78.6 (0.4)	-2.0 (0.4)	-
**P16-A**	119.2 (84.3 – 168.6)	13	96 (± 4)	78.9 (0.2)	-1.7 (0.2)	-
**P17-A**	66.7 (43.7 – 101.7)	7	98 (± 3)	79.5 (0.1)	-1.1 (0.2)	-
**P18-A**	77.7 (28.2 – 213.7)	9	100 (± 7)	80.3 (0.2)	-0.3 (0.3)	-
**P19-A**	63.3 (22.1 – 181.3)	7	92 (± 6)	79.9 (0.6)	-0.7 (0.6)	-
**P20-A**	27.9 (20.3 – 38.3)	3	88 (± 5)	80.4 (0.3)	-0.2 (0.3)	-
**3'-A**	16.8 (16.0 – 17.5)	2	92 (± 4)	80.4 (0.1)	-0.2 (0.2)	10.1 (6.2 – 16.7)
**3'-B**	356.7 (70.0 – 1817.8)	37	109 (± 12)	79.8 (1.5)	-0.8 (1.5)	9.2 (6.6 – 12.9)
**3'-C**	>10^5	n/a	n/a	-	-	-
**NTC-3'-A**	19.4 (7.5 – 50.2)	2	93 (± 2)	81.9 (0.1)	1.3 (0.1)	-
**C5-10-A**	-	-	-	76.8 (0.5)	-3.8 (0.5)	25.8 (11.6 – 63.2)
**2'-10-A**	-	-	-	80.3 (0.2)	-0.3 (0.2)	47.9 (21.4 – 112.6)
Free peptides						
**A**	9.0 (8.8 – 9.3)	1	88 (± 4)	-	-	-
**B**	11.1 (6.2 – 19.9)	1	93 (± 3)	-	-	-
**C**	>10^5	n/a	n/a	-	-	-
Free siRNA						
**HPRT1 siRNA**	-	-	-	80.6 (0.1)	0 (0)	6.0 (4.1 - 8.7)
**NTC siRNA**	-	-	-	82.1 (0.2)	1.5 (0.3)	n/a

Supplementary Figure S4. Data table from GLP1R activation, melting studies, and HPRT1 knockdown.

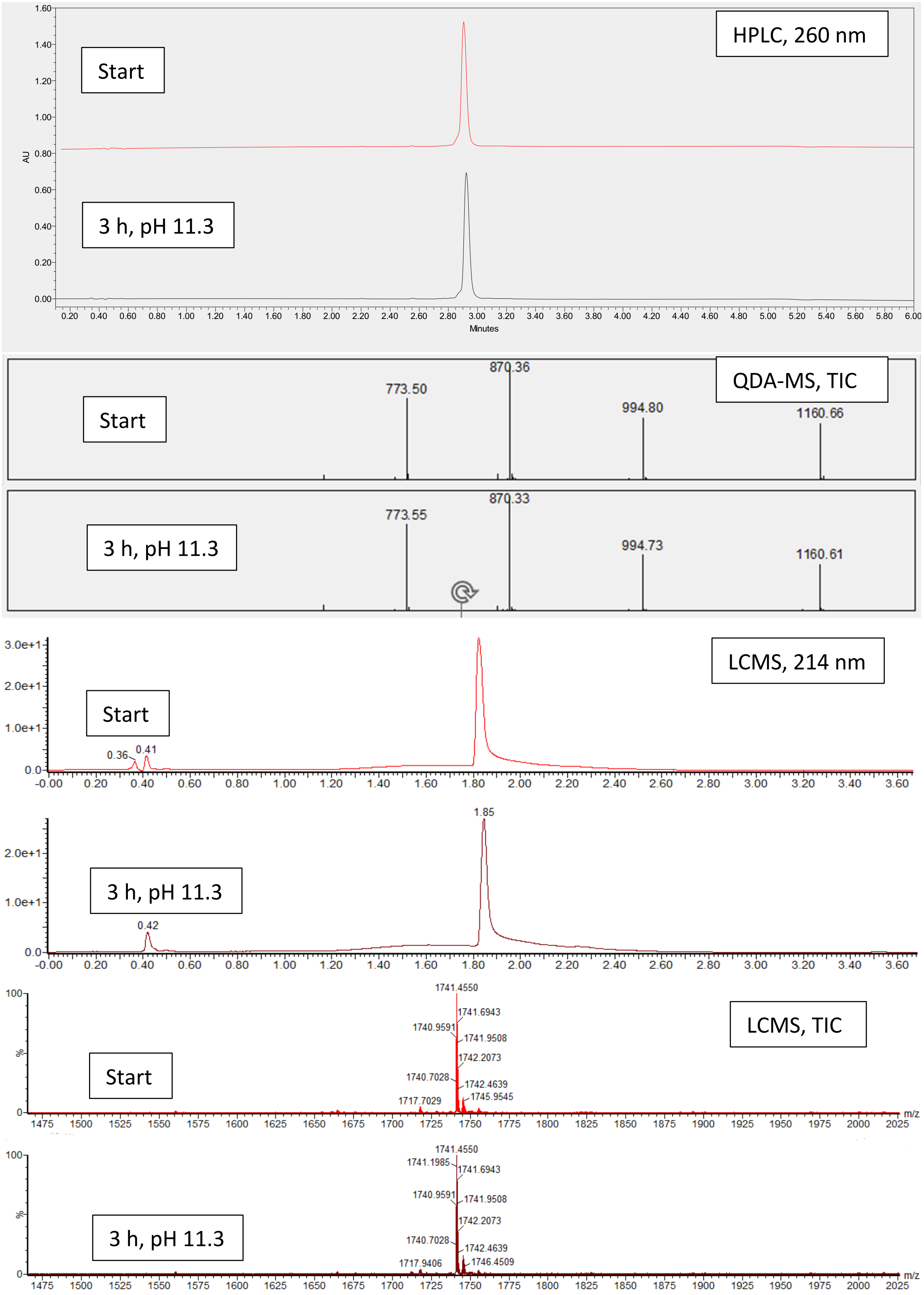

Supplementary Figure S5. Oligonucleotide pH-stability analysis. Amino-modified oligonucleotide (SS HPRT1 3'-amino) was incubated at pH 11.3 for 3 h and analyzed by HPLC Method B and LCMS Method A.

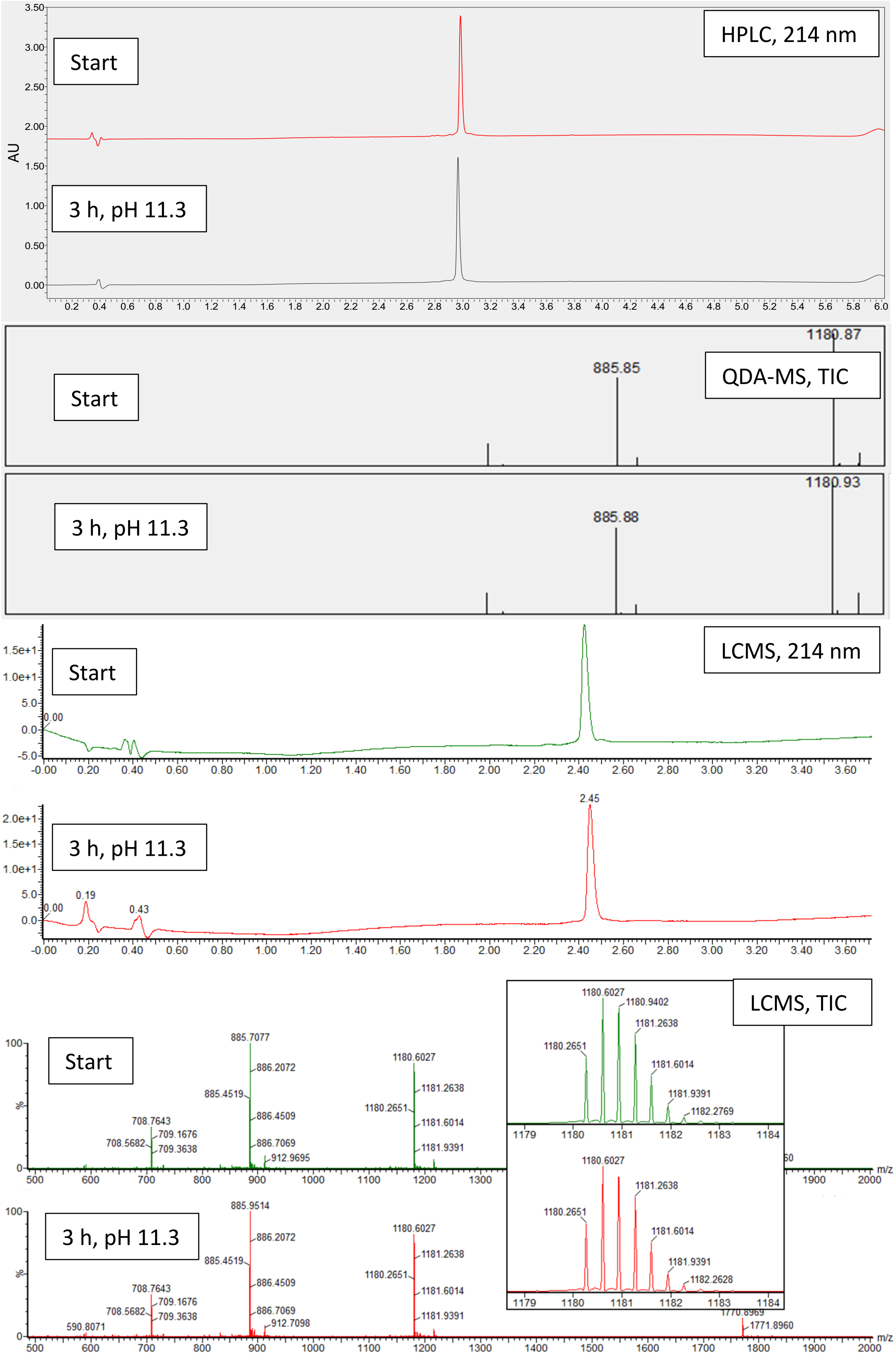

Supplementary Figure S6. Peptide stability analysis. Peptide A was incubated at pH 11.3 for 3 h and analyzed by HPLC Method G and LCMS Method B.

Synthesis data

All reagents and materials used were purchased from ordinary chemical suppliers and were used without further purification. The solvents used were of standard HPLC grade. ^{1}H NMR spectra were acquired on a Bruker Aeon-400 MHz spectrometer at ambient temperature. ^{13}C NMR spectra were recorded at 100 MHz with complete proton decoupling. Samples were prepared in deuterated dimethyl sulfoxide (DMSO-d_6) or chloroform $CDCl_3$. Chemical shifts are reported in ppm (δ) relatively to the chemical shift of the deuterated solvent. Multiplicity is reported as follows: singlet (s), doublet (d), doublet of doublets (dd), triplet (t), quartet (q), quintet (qt) and multiplet (m). Masses were confirmed by either liquid chromatography mass spectrometry (LCMS) or high-resolution mass spectrometry (HRMS). LCMS was recorded using atmospheric pressure ionization electrospray (API-ES) on a Waters Xevo G2-XS QToF, and HRMS analysis was performed on a Bruker Daltonics micrOTOF.

2-(1,3-Dioxoisoindolin-2-yl)ethane-1-sulfonyl azide (**1**)

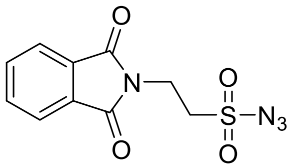

In a 500 mL round-bottomed flask equipped with a magnetic stirrer bar, 2-phthalimidoethanesulfonyl chloride (50.0 g, 183 mmol, 1 eq.) and sodium azide (14.3 g, 219 mmol, 1.2 eq.) were suspended in acetone (160 mL) and water (40 mL). The stirred suspension was cooled on an ice bath. Via an addition funnel, triethylamine (31.8 mL, 183 mmol, 1 eq.) was added over 20 min, at which point the mixture had turned into a solution and UPLC analysis indicated full conversion of sulfonyl chloride. The reaction mixture was poured into water (800 mL), and stirred for 20 min. The solid was filtered off and transferred to a 2 L conical flask containing a magnet. The solid was dissolved in minimal boiling ethanol (~500 mL). Upon slow cooling to room temperature, the product crystalized out of solution as colorless needles. The mother liquor was filtered off and the crystals were washed with ice-cold ethanol and dried under vacuum overnight to form 33 g colorless needles. 64% yield. ^{1}H-NMR (400 MHz, $CDCl_3$) δ ppm 7.98 (dd, J = 5.48, 3.04 Hz, 2H), 7.75 (dd, J = 5.48, 3.04 Hz, 2H), 4.23 (t, J = 6.46 Hz, 2H), 3.73 (t, J = 6.46 Hz, 2H). ^{13}C-NMR (100 MHz, $CDCl_3$) δ ppm 167.6 (2C), 134.6 (2C), 131.8 (2C), 123.8 (2C), 52.4, 32.3. HRMS, m/z calculated for $C_{10}H_8N_4O_4SNa$ $[M+Na]^+$ 303.0158, found 303.0155.

3,5-Dichloro-2-((12-((2,5-dioxopyrrolidin-1-yl)oxy)-12-oxododecanoyl)oxy)benzenesulfonic acid (**2**)

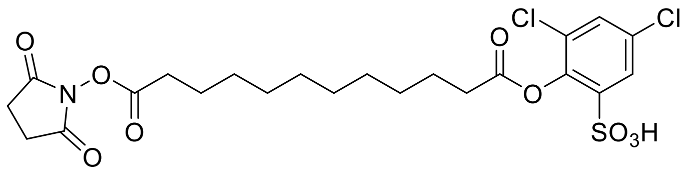

To a solution of mono-tert-butyl dodecanedioic acid (2.86 g, 10.0 mmol, 1 eq.) in DCM (20 mL) was added triethylamine (2.09 mL, 15.0 mmol, 1.5 eq.), followed by 3,5-dichloro-2-hydroxybenzenesulfonyl chloride (2.75 g, 10.5 mmol, 1.05 eq.). The mixture was stirred for 1.5 h at room temperature, then quenched by

5 w/w% $NaHSO_4$ (aq.) (50 mL), and extracted with EtOAc (50 mL). The organic phase was washed with $NaHCO_3$ (aq. sat.)/brine 1:1 (50 mL). 2-Propanol (10 mL) was added to aid phase separation. The organic phase was washed with 1 M HCl (aq.) (50 mL), followed by brine (50 mL), and dried over Na_2SO_4. The solvent was removed under reduced pressure. The residue was dissolved in DCM (20 mL), and cooled on an ice bath. While stirring, TFA (20 mL) was added. The ice bath was removed and the reaction was stirred at room temperature for 2 h. The reaction mixture was poured into ice-cold (0 °C) diethylether (200 mL), and the precipitate was isolated by centrifugation. The precipitate was suspended and centrifuged twice in diethylether (2 x 100 mL), and then dried under a stream of nitrogen. The precipitate was dissolved in acetone (20 mL), and stirred at room temperature when *N*-hydroxysuccinimide (2.30 g, 20.0 mmol, 2 eq.) was added. Then EDC*HCl (2.88 g, 15.0 mmol, 1.5 eq.) was added, and the mixture was stirred for 5 h at 30 °C. The mixture was diluted with EtOAc (100 mL) and washed with 0.1 M HCl (aq.) (3 x 50 mL), followed by brine (50 mL), and finally dried over Na_2SO_4. The solvent volume was reduced under reduced pressure to ~10 mL, and the product was precipitated with ice-cold diethyl ether. The mother liquor was decanted off and the precipitate was redissolved in minimal acetone and precipitated from ice-cold diethylether. The mother liquor was filtered off and the product was dried under vacuum overnight to afford a white solid (2.50 g) in 45% yield. ^{1}H-NMR (400 MHz, DMSO-d$_6$) δ ppm 7.72 (d, *J* = 2.52 Hz, 1H), 7.65 (d, *J* = 2.52 Hz, 1H), 2.81 (s, 4H), 2.65 (t, *J* = 7.18 Hz, 2H), 2.58-2.51 (m, 2H), 1.62 (dqt, *J* = 7.19, 1.56 Hz, 4H), 1.41-1.24 (m, 12H). ^{13}C-NMR (100 MHz, DMSO-d$_6$) δ ppm 170.3 (2C), 169.7, 169.0, 143.3, 142.7, 129.7, 129.5, 128.8, 126.8, 33.4, 30.2, 28.8, 28.8, 28.7, 28.5, 28.4, 28.0, 25.4 (2C), 24.3, 24.0. LCMS Method C: Rt 3.14 min, m/z calculated for $C_{22}H_{26}Cl_2NO_9S$ [M-H]$^-$ 550.0711, found 550.0775.

2,5-Dioxopyrrolidin-1-yl tetradecanoate (**3**)

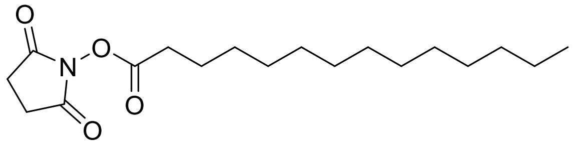

To a stirred solution of myristic acid (228 mg, 1.00 mmol, 1 eq.) in acetone (2 mL) was added *N*-hydroxysuccinimide (132 mg, 1.15 mmol, 1.15 eq.) followed by EDC*HCl (211 mg, 1.10 mmol, 1.1 eq.). The mixture was stirred for 5 h at 30 °C. The mixture was diluted with EtOAc (10 mL) and washed with 0.1 M HCl (aq.) (2 x 10 mL), followed by brine (10 mL), and finally dried over Na_2SO_4. The solvent was removed under reduced pressure. The product was recrystallized from 2-propanol, then dried under vacuum overnight to afford colorless crystals (260 mg) in 80% yield. ^{1}H-NMR (400 MHz, $CDCl_3$) δ ppm 2.82 (s, 4H), 2.59 (t, *J* = 7.50 Hz, 2H), 1.73 (qt, *J* = 7.50 Hz, 2H), 1.39 (qt, *J* = 7.28 Hz, 2H), 1.34-1.19 (m, 18H), 0.87 (t, *J* = 6.82 Hz, 3H). ^{13}C-NMR (100 MHz, $CDCl_3$) δ ppm 169.4 (2C), 138.9, 32.1, 31.2, 29.9, 29.9, 29.8, 29.8, 29.6 (2C), 29.3, 29.0, 25.8 (2C), 24.8, 22.9, 14.3. LCMS Method C: Rt 4.58 min, m/z calculated for $C_{18}H_{30}NO_4$ [M-H]$^-$ 324.2180, found 324.2180.

3,5-Dichloro-2-(tetradecanoyloxy)benzenesulfonic acid (**4**)

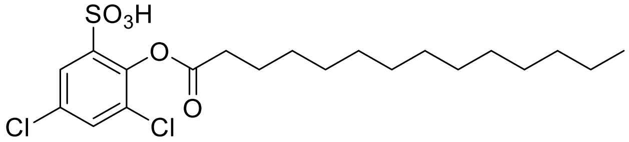

To a stirred solution of myristic acid (228 mg, 1.00 mmol, 1 eq.) in 2-MeTHF (2 mL) was added triethylamine (209 µL, 1.50 mmol, 1.5 eq.), followed by 3,5-dichloro-2-hydroxybenzenesulfonyl chloride (288 mg, 1.10 mmol, 1.1 eq.). The mixture was stirred for 2 h at room temperature. The mixture was then diluted with EtOAc (10 mL) and filtered before washing with 5 w/w% $NaHSO_4$ (aq.) (10 mL), then with $NaHCO_3$ (aq. sat.)/brine 1:1 (10 mL). 2-Propanol (2 mL) was added to aid phase separation. The organic phase was washed with 1 M HCl (aq.) (2 x 10 mL), followed by brine (10 mL), and dried over Na_2SO_4 and concentrated. The residue was purified by flash chromatography eluting with a gradient from DCM to 20% methanol in DCM to afford the product as a white solid (339 mg), in 75% yield. ^{1}H-NMR (400 MHz, DMSO-d_6) δ ppm 7.72 (d, *J* = 2.58 Hz, 1H), 7.64 (d, *J* = 2.58 Hz, 1H), 2.56-2.49 (m, 2H), 1.62 (qt, *J* = 7.60 Hz, 2H), 1.41-1.19 (m, 20H), 0.85 (t, *J* = 6.8 Hz, 3H). ^{13}C-NMR (100 MHz, DMSO-d_6) δ ppm 169.7, 143.3, 142.7, 129.6, 129.5, 128.8, 126.8, 33.4, 31.3, 29.1, 29.0, 29.0, 29.0, 28.9, 28.7 (2C), 28.4, 24.0, 22.1, 14.0. LCMS Method A: Rt 4.24 min, m/z calculated for $C_{20}H_{29}Cl_2O_5S$ $[M-H]^-$ 451.1118, found 451.1255.

HPLC methods

HPLC Method A: Solvent A: 1% HFIP, 0.3% TEA in water. Solvent B: MeOH. Gradient: Linear gradient from 10-30% B over 40 min. UV detection: 260 nm. Column: Phenomenex Gemini 5 µm NX-C18 110 Å, 250 x 21.2 mm. System: Waters LC prep purification system.

HPLC Method B: Solvent A: 400 mM HFIP, 15 mM TEA in water. Solvent B: 400 mM HFIP, 15 mM TEA in MeOH. Gradient: Linear gradient from 10-40% B over 5 min. UV detection: 260 nm. Column: Waters Acquity BEH C18 130 Å 1.7 µm, 2.1 x 150 mm. Temperature: 60 °C. System: Waters Acquity UPLC system with Acquity QDa mass detector. Ionization mode: Negative (API-ES-).

HPLC Method C: Solvent A: 0.1% TFA in water. Solvent B: 0.1% TFA in acetonitrile. Gradient: Linear gradient from 30-55% B over 40 min. UV detection: 214 nm. Column: Phenomenex Gemini 5 µm NX-C18 110 Å, 250 x 30 mm. System: Waters LC prep purification system.

HPLC Method D: Solvent A: 0.1 M TEAA in water. Solvent B: Acetonitrile. Gradient: Linear gradient from 20-40% B over 15 min. UV detection: 260 nm. Column: Phenomenex Clarity 5µm Oligo-RP, 100 x 10 mm. System: Agilent 1200 Infinity LC system.

HPLC Method E: Solvent A: 0.1 M TEAA in water. Solvent B: Acetonitrile. Gradient: Linear gradient from 15-30% B over 15 min. UV detection: 260 nm. Column: Phenomenex Clarity 5 µm Oligo-RP, 100 x 10 mm. System: Agilent 1200 Infinity LC system.

HPLC Method F: Solvent A: 400 mM HFIP, 15 mM TEA in water. Solvent B: 400 mM HFIP, 15 mM TEA in MeOH. Gradient: Linear gradient from 20-50% B over 5 min. UV detection: 260 nm. Column: Waters Acquity BEH C18 130Å 1.7 µm, 2.1 x 150 mm. Temperature: 60° C. System: Waters Acquity UPLC system with Acquity QDa mass detector. Ionization mode: Negative (API-ES-).

HPLC Method G: Solvent A: 0.1% TFA in water. Solvent B: 0.1% TFA in acetonitrile. Gradient: Linear gradient from 5-60% B over 4 min. UV detection: 214 nm. Column: Waters Acquity BEH C18 130 Å 1.7 µm, 2.1 x 150 mm. Temperature: 40 °C. System: Waters Acquity UPLC system with Acquity QDa mass detector. Ionization mode: Positive (API-ES+).

LCMS methods

LCMS Method A: Solvent A: Water. Solvent B: Acetonitrile. Solvent C: 100 mM triethylammonium acetate in water:acetonitrile 1:1, pH 7.8. Gradient: 2.5% C + linear gradient from 0-97.5% B over 4.5 min. UV detection: 214 nm. Column: Waters Acquity BEH C18 130 Å 1.7 µm, 2.1 x 50 mm. Temperature: 40 °C. System: Waters Xevo G2-XS QToF. Ionization mode: Negative (API-ES-).

LCMS Method B: Solvent A: Water. Solvent B: Acetonitrile. Solvent C: 2% formic acid + 0.1% TFA in water. Gradient: 5% C + linear gradient from 5-90% B over 4 min. UV detection: 214 nm. Column: Waters Acquity BEH C18 130 Å 1.7 µm, 2.1 x 50 mm. Temperature: 40 °C. System: Waters Xevo G2-XS QToF. Ionization mode: Positive (API-ES+).

LCMS Method C: Solvent A: Water. Solvent B: Acetonitrile. Solvent C: 50 mM ammonium formate in water, pH 9. Gradient: 5% C + linear gradient from 0-95% B over 4 min. UV detection: 214 nm. Column: Waters Acquity BEH C18 130 Å 1.7 µm, 2.1 x 50 mm. Temperature: 40 °C. System: Waters Xevo G2-XS QToF. Ionization mode: Negative (API-ES-).

Compound mass analysis

All ONs, peptides, and peptide-ON conjugates were analyzed by QDa mass detection during HPLC analysis and final conjugates were also analyzed by LCMS. Mass detection with QDa was done with HPLC method B for unmodified and amino-modified ONs, and with HPLC method F for DCSP-modified and peptide-conjugated ONs. For LCMS analysis, ONs and conjugates were both analyzed by LCMS method A. Free peptides were analyzed by HPLC method G and LCMS method B.

**Compound**	**Calc. molar mass (Da)**	**QDa found ion**	**QDa calcd. ion mass (Da)**	**QDa found mass (Da)**	**LCMS deconv. found mass (Da)**
**Antisense strands**					
AS HPRT1	7682.0	$[M-H_7]^{-7}$	1096.4	1096.3	7682
AS NTC	7739.0	$[M-H_7]^{-7}$	1104.6	1104.6	7739
**Sense strands**					
SS HPRT1	6791.2	$[M-H_7]^{-7}$	969.2	969.2	6791
SS NTC	6757.3	$[M-H_8]^{-8}$	843.7	843.7	6757
**Amino-modified sense strands**					
SS HPRT1 5'-amino	6970.4	$[M-H_6]^{-6}$	1160.7	1160.7	6971
SS HPRT1 P1-amino	6881.3	$[M-H_6]^{-6}$	1145.9	1145.8	-
SS HPRT1 P2-amino	6881.3	$[M-H_7]^{-7}$	982.0	982.0	-
SS HPRT1 P3-amino	6897.4	$[M-H_6]^{-6}$	1148.6	1148.4	-
SS HPRT1 P4-amino	6897.4	$[M-H_7]^{-7}$	984.3	984.3	-
SS HPRT1 P5-amino	6897.4	$[M-H_6]^{-6}$	1148.6	1148.5	-
SS HPRT1 P6-amino	6897.4	$[M-H_7]^{-7}$	984.3	984.4	-

SS HPRT1 P7-amino	6897.4	$[M-H_6]^{-6}$	1148.6	1148.5	-
SS HPRT1 P8-amino	6897.4	$[M-H_7]^{-7}$	984.3	984.4	-
SS HPRT1 P9-amino	6897.4	$[M-H_6]^{-6}$	1148.6	1148.4	-
SS HPRT1 P10-amino	6897.4	$[M-H_6]^{-6}$	1148.6	1148.5	-
SS HPRT1 P11-amino	6897.4	$[M-H_7]^{-7}$	984.3	984.4	-
SS HPRT1 P12-amino	6897.4	$[M-H_6]^{-6}$	1148.6	1148.5	-
SS HPRT1 P13-amino	6897.4	$[M-H_6]^{-6}$	1148.6	1148.6	-
SS HPRT1 P14-amino	6897.4	$[M-H_6]^{-6}$	1148.6	1148.5	-
SS HPRT1 P15-amino	6897.4	$[M-H_6]^{-6}$	1148.6	1148.5	-
SS HPRT1 P16-amino	6897.4	$[M-H_7]^{-7}$	984.3	984.4	-
SS HPRT1 P17-amino	6897.4	$[M-H_7]^{-7}$	984.3	984.3	-
SS HPRT1 P18-amino	6897.4	$[M-H_7]^{-7}$	984.3	984.3	-
SS HPRT1 P19-amino	6897.4	$[M-H_6]^{-6}$	1148.6	1148.5	-
SS HPRT1 P20-amino	6897.4	$[M-H_6]^{-6}$	1148.6	1148.5	-
SS HPRT1 3’-amino	6970.4	$[M-H_6]^{-6}$	1160.7	1160.7	6971
SS NTC 3’-amino	6936.4	$[M-H_6]^{-6}$	1155.1	1155.2	6937
SS HPRT1 C5-10-amino	6873.3	$[M-H_6]^{-6}$	1144.6	1144.4	6873
SS HPRT1 2’-10-amino	6834.3	$[M-H_7]^{-7}$	975.3	975.3	6833
**DCSP-modified sense strands**					
SS HPRT1 5’-DCSP	7407.7	$[M-H_7]^{-7}$	1057.2	1057.3	-
SS HPRT1 P1-DCSP	7318.6	$[M-H_7]^{-7}$	1044.5	1044.5	-
SS HPRT1 P2-DCSP	7318.6	$[M-H_6]^{-6}$	1218.8	1218.7	-
SS HPRT1 P3-DCSP	7334.7	$[M-H_7]^{-7}$	1046.8	1046.8	-
SS HPRT1 P4-DCSP	7334.7	$[M-H_7]^{-7}$	1046.8	1046.8	-
SS HPRT1 P5-DCSP	7334.7	$[M-H_7]^{-7}$	1046.8	1046.8	-
SS HPRT1 P6-DCSP	7334.7	$[M-H_7]^{-7}$	1046.8	1046.8	-
SS HPRT1 P7-DCSP	7334.7	$[M-H_7]^{-7}$	1046.8	1046.8	-
SS HPRT1 P8-DCSP	7334.7	$[M-H_6]^{-6}$	1221.5	1221.4	-
SS HPRT1 P9-DCSP	7334.7	$[M-H_7]^{-7}$	1046.8	1046.8	-
SS HPRT1 P10-DCSP	7334.7	$[M-H_6]^{-6}$	1221.5	1221.4	-
SS HPRT1 P11-DCSP	7334.7	$[M-H_6]^{-6}$	1221.5	1221.4	-
SS HPRT1 P12-DCSP	7334.7	$[M-H_7]^{-7}$	1046.8	1046.8	-
SS HPRT1 P13-DCSP	7334.7	$[M-H_7]^{-7}$	1046.8	1046.7	-
SS HPRT1 P14-DCSP	7334.7	$[M-H_6]^{-6}$	1221.5	1221.3	-
SS HPRT1 P15-DCSP	7334.7	$[M-H_6]^{-6}$	1221.5	1221.4	-
SS HPRT1 P16-DCSP	7334.7	$[M-H_7]^{-7}$	1046.8	1046.7	-
SS HPRT1 P17-DCSP	7334.7	$[M-H_7]^{-7}$	1046.8	1046.7	-
SS HPRT1 P18-DCSP	7334.7	$[M-H_8]^{-8}$	915.8	915.7	-
SS HPRT1 P19-DCSP	7334.7	$[M-H_7]^{-7}$	1046.8	1046.8	-
SS HPRT1 P20-DCSP	7334.7	$[M-H_7]^{-7}$	1046.8	1046.9	-
SS HPRT1 3’-DCSP	7407.7	$[M-H_8]^{-8}$	925.0	925.1	-
SS NTC 3’-DCSP	7373.7	$[M-H_7]^{-7}$	1052.4	1052.4	-
SS HPRT1 C5-10-DCSP	7310.7	$[M-H_7]^{-7}$	1043.4	1043.3	-
SS HPRT1 2’-10-DCSP	7271.6	$[M-H_7]^{-7}$	1037.8	1037.7	-
**Peptide-modified sense strands**					

SS HPRT1 5'-B	10548.4	$[M-H_9]^{-9}$	1171.0	1171.0	10549
SS HPRT1 5'-A	10704.6	$[M-H_9]^{-9}$	1188.4	1188.3	10705
SS HPRT1 P1-A	10615.5	$[M-H_9]^{-9}$	1178.5	1178.5	10616
SS HPRT1 P2-A	10615.5	$[M-H_9]^{-9}$	1178.5	1178.5	10616
SS HPRT1 P3-A	10631.6	$[M-H_9]^{-9}$	1180.3	1180.1	10632
SS HPRT1 P4-A	10631.6	$[M-H_9]^{-9}$	1180.3	1180.2	10632
SS HPRT1 P5-A	10631.6	$[M-H_9]^{-9}$	1180.3	1180.2	10632
SS HPRT1 P6-A	10631.6	$[M-H_9]^{-9}$	1180.3	1180.2	10632
SS HPRT1 P7-A	10631.6	$[M-H_9]^{-9}$	1180.3	1180.2	10632
SS HPRT1 P8-A	10631.6	$[M-H_9]^{-9}$	1180.3	1180.1	10632
SS HPRT1 P9-A	10631.6	$[M-H_9]^{-9}$	1180.3	1180.2	10632
SS HPRT1 P10-A	10631.6	$[M-H_9]^{-9}$	1180.3	1180.0	10632
SS HPRT1 P11-A	10631.6	$[M-H_9]^{-9}$	1180.3	1180.2	10632
SS HPRT1 P12-A	10631.6	$[M-H_9]^{-9}$	1180.3	1180.2	10632
SS HPRT1 P13-A	10631.6	$[M-H_9]^{-9}$	1180.3	1180.3	10632
SS HPRT1 P14-A	10631.6	$[M-H_9]^{-9}$	1180.3	1180.0	10632
SS HPRT1 P15-A	10631.6	$[M-H_9]^{-9}$	1180.3	1180.0	10632
SS HPRT1 P16-A	10631.6	$[M-H_9]^{-9}$	1180.3	1180.2	10632
SS HPRT1 P17-A	10631.6	$[M-H_9]^{-9}$	1180.3	1180.1	10632
SS HPRT1 P18-A	10631.6	$[M-H_9]^{-9}$	1180.3	1180.1	10632
SS HPRT1 P19-A	10631.6	$[M-H_9]^{-9}$	1180.3	1180.3	10632
SS HPRT1 P20-A	10631.6	$[M-H_9]^{-9}$	1180.3	1180.1	10632
SS HPRT1 3'-A	10704.6	$[M-H_9]^{-9}$	1188.4	1188.4	10705
SS HPRT1 3'-B	10548.4	$[M-H_9]^{-9}$	1171.0	1171.0	10549
SS HPRT1 3'-C	10704.6	$[M-H_9]^{-9}$	1188.4	1188.3	10705
SS NTC 3'-A	10670.6	$[M-H_9]^{-9}$	1184.6	1184.6	10671
SS HPRT1 C5-10-A	10607.5	$[M-H_9]^{-9}$	1177.6	1177.4	10608
SS HPRT1 2'-10-A	10568.5	$[M-H_9]^{-9}$	1173.3	1173.3	10569
**Peptides**					
A	3539.9	$[M+H_3]^{+3}$	1181.0	1180.8	3540
B	3383.7	$[M+H_3]^{+3}$	1128.9	1128.8	3385
C	3539.9	$[M+H_3]^{+3}$	1181.0	1180.7	3540

NMR spectra

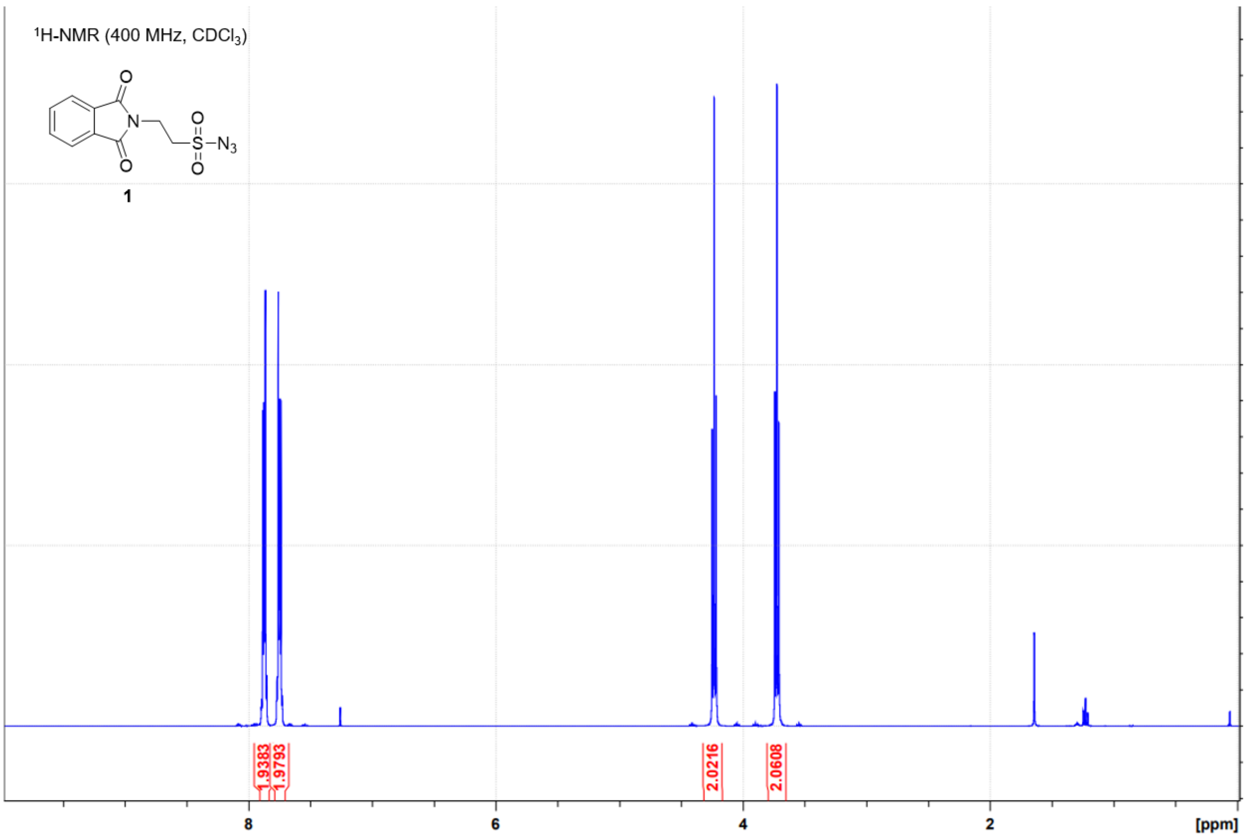

1H-NMR (400 MHz, CDCl3)
O
N
O
S
N3
O
O
1
1.9383
1.9793
2.0216
2.0608
8
6
4
2
[ppm]

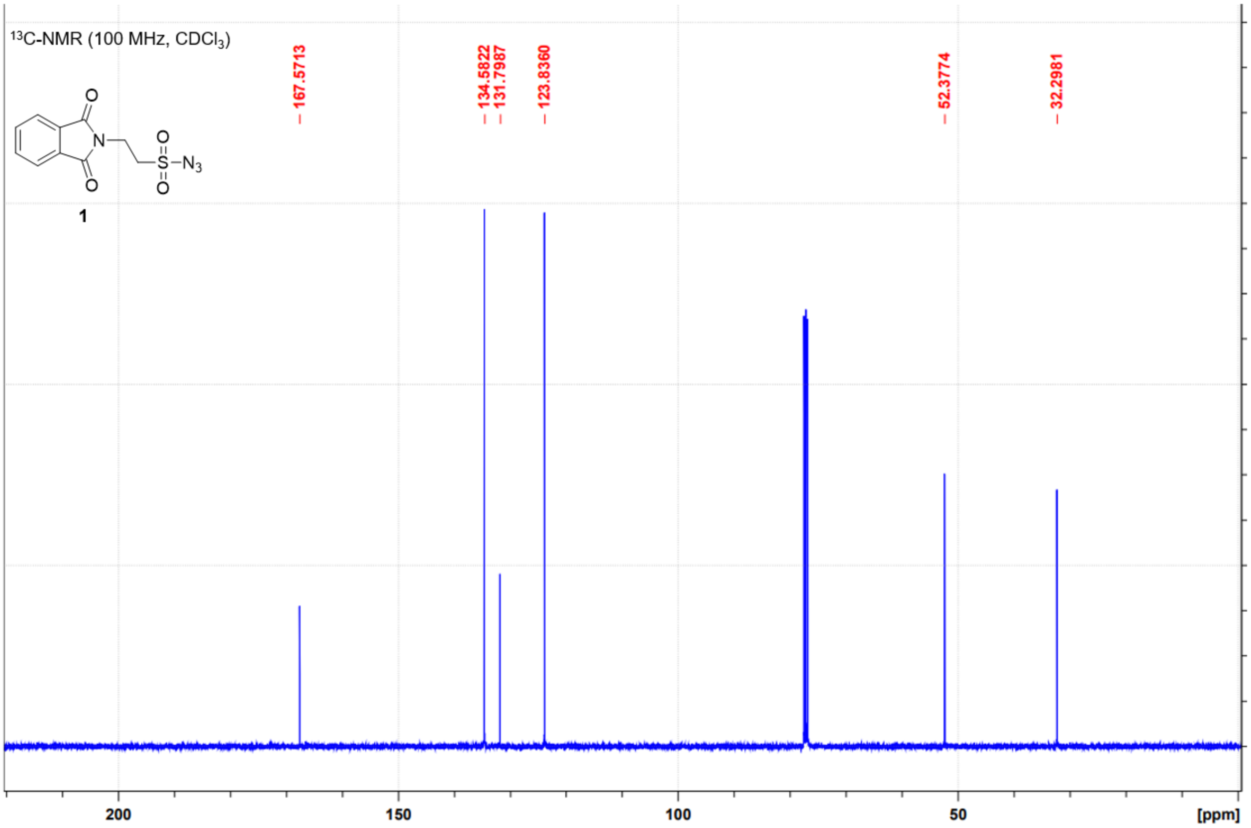

13C-NMR (100 MHz, CDCl3)
167.5713
134.5822
131.7987
123.8360
52.3774
32.2981
O
N
O
S
N3
O
O
1
200
150
100
50
[ppm]

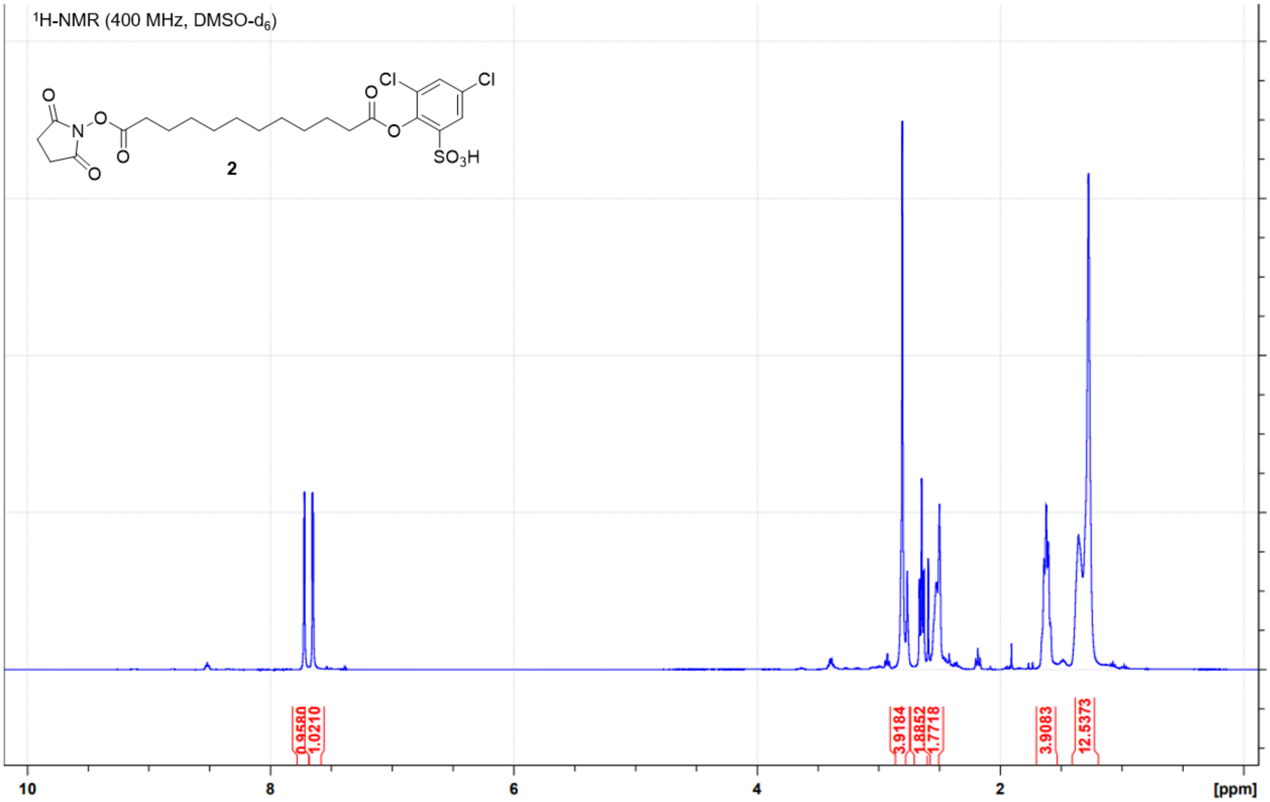
1H-NMR (400 MHz, DMSO-d6)
Cl
Cl
O
O
N
O
O
O
O
SO3H
2
0.9580
1.0210
3.9184
1.8852
1.7718
3.9083
12.5373
10
8
6
4
2
[ppm]

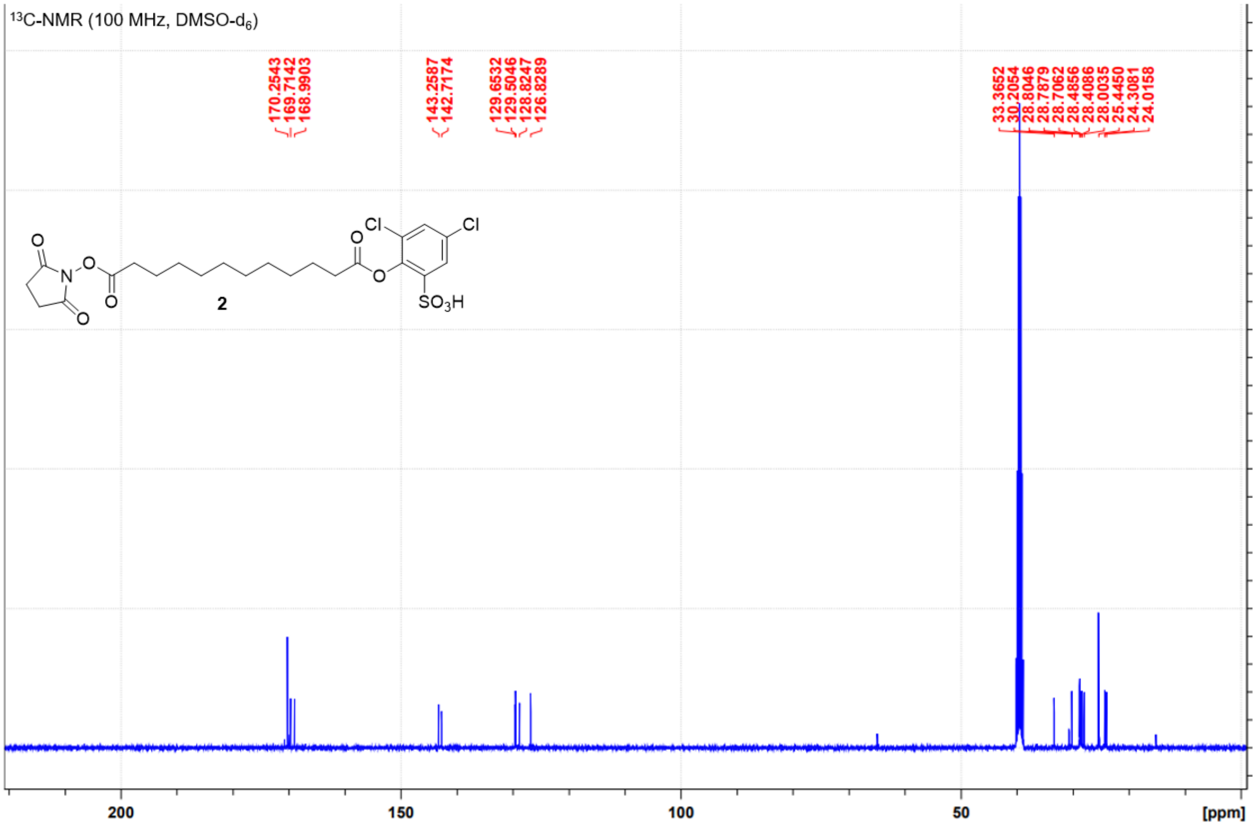
13C-NMR (100 MHz, DMSO-d6)
170.2543
169.7142
168.9903
143.2587
142.7174
129.6532
129.5046
128.8247
126.8289
33.3652
30.2054
28.8046
28.7879
28.7062
28.4856
28.4086
28.0035
25.4450
24.3081
24.0158
Cl
Cl
O
O
N
O
O
O
O
SO3H
2
200
150
100
50
[ppm]

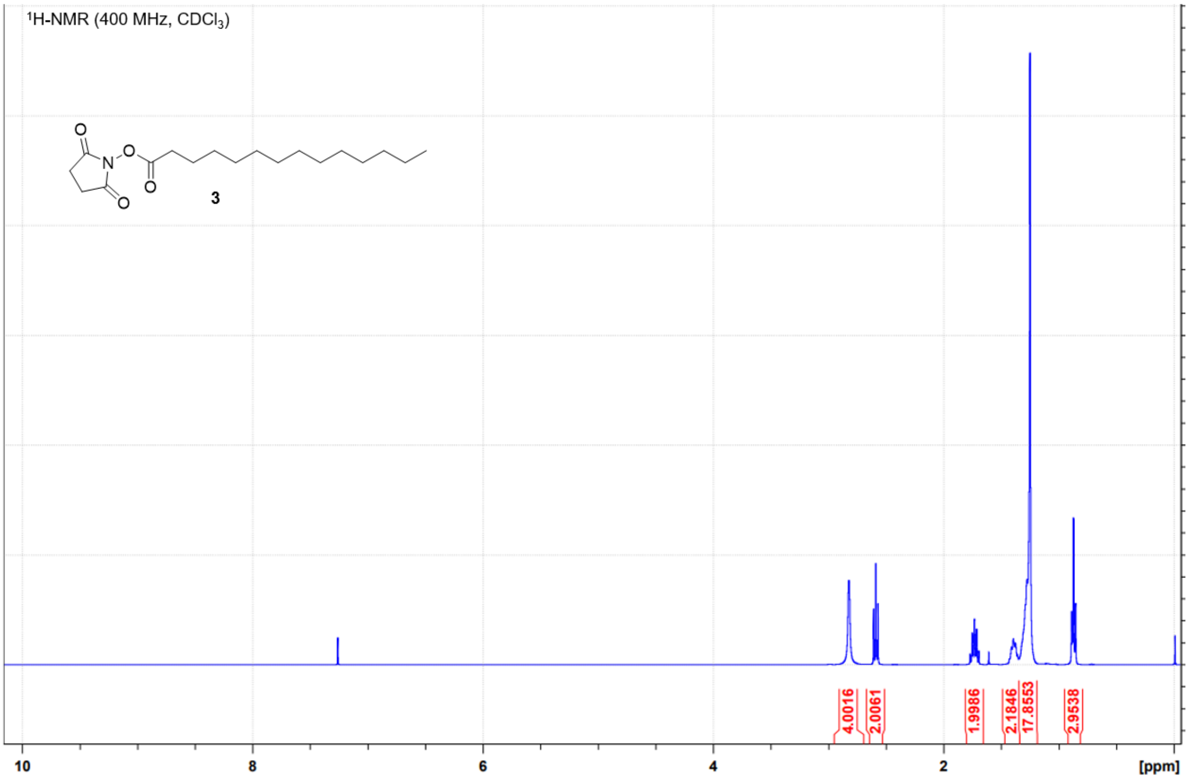

1H-NMR (400 MHz, CDCl3)
3
4.0016
2.0061
1.9986
2.1846
17.8553
2.9538
10
8
6
4
2
[ppm]

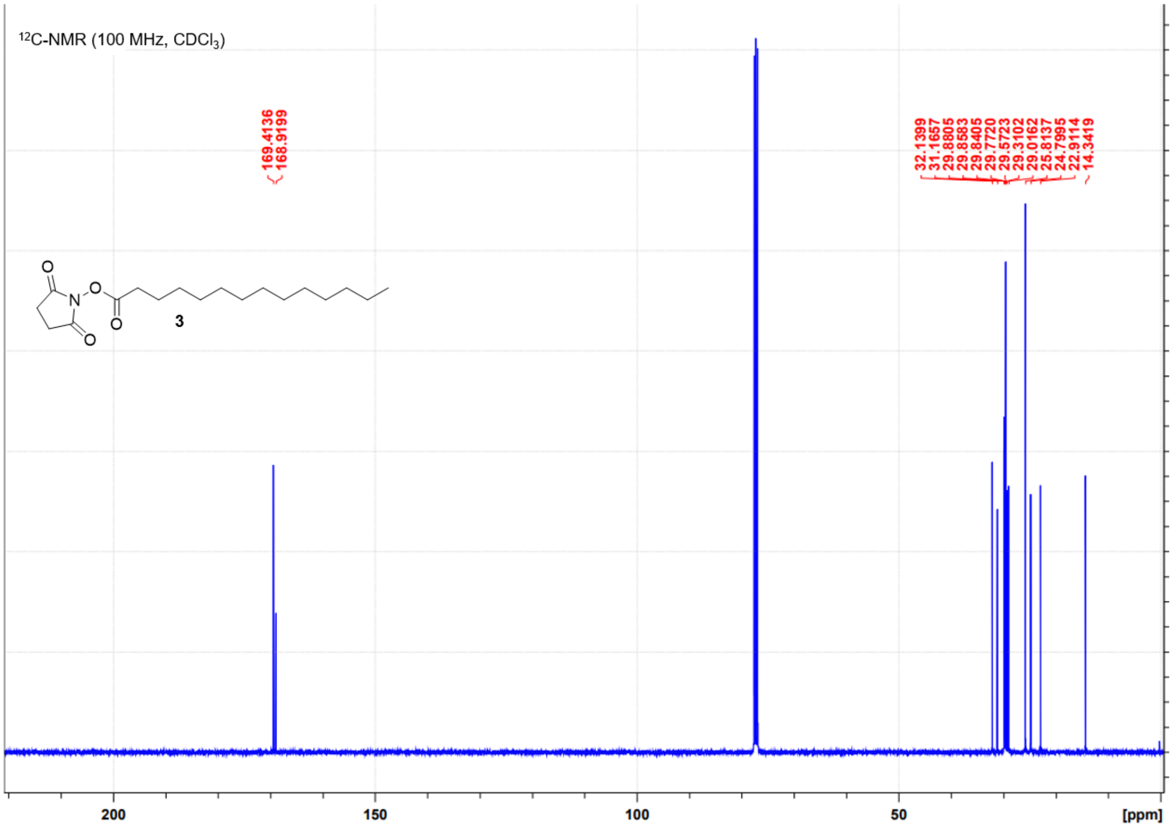

12C-NMR (100 MHz, CDCl3)
169.4136
168.9199
32.1399
31.1657
29.8805
29.8583
29.8405
29.7720
29.5723
29.3102
29.0162
25.8137
24.7995
22.9114
14.3419
3
200
150
100
50
[ppm]

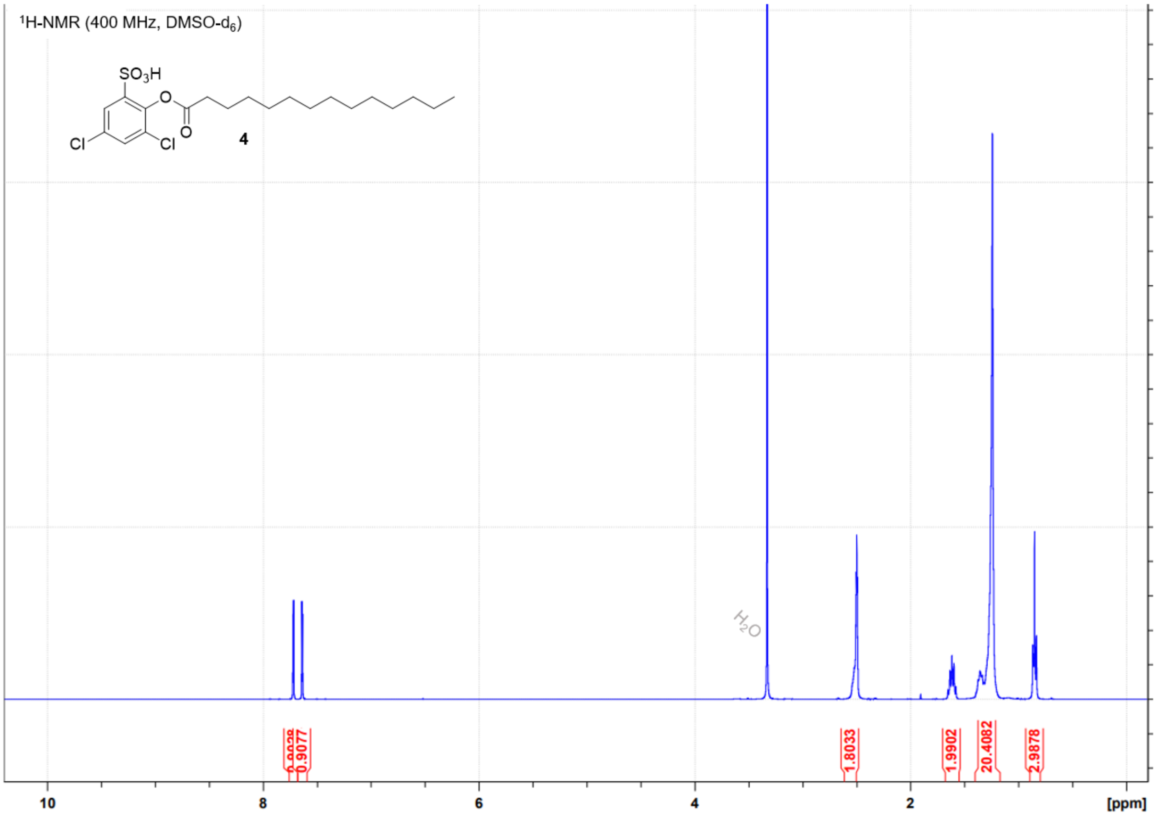

1H-NMR (400 MHz, DMSO-d6)
SO3H
O
O
Cl
Cl
4
H2O
0.9077
1.8033
1.9902
20.4082
2.9878
10
8
6
4
2
[ppm]

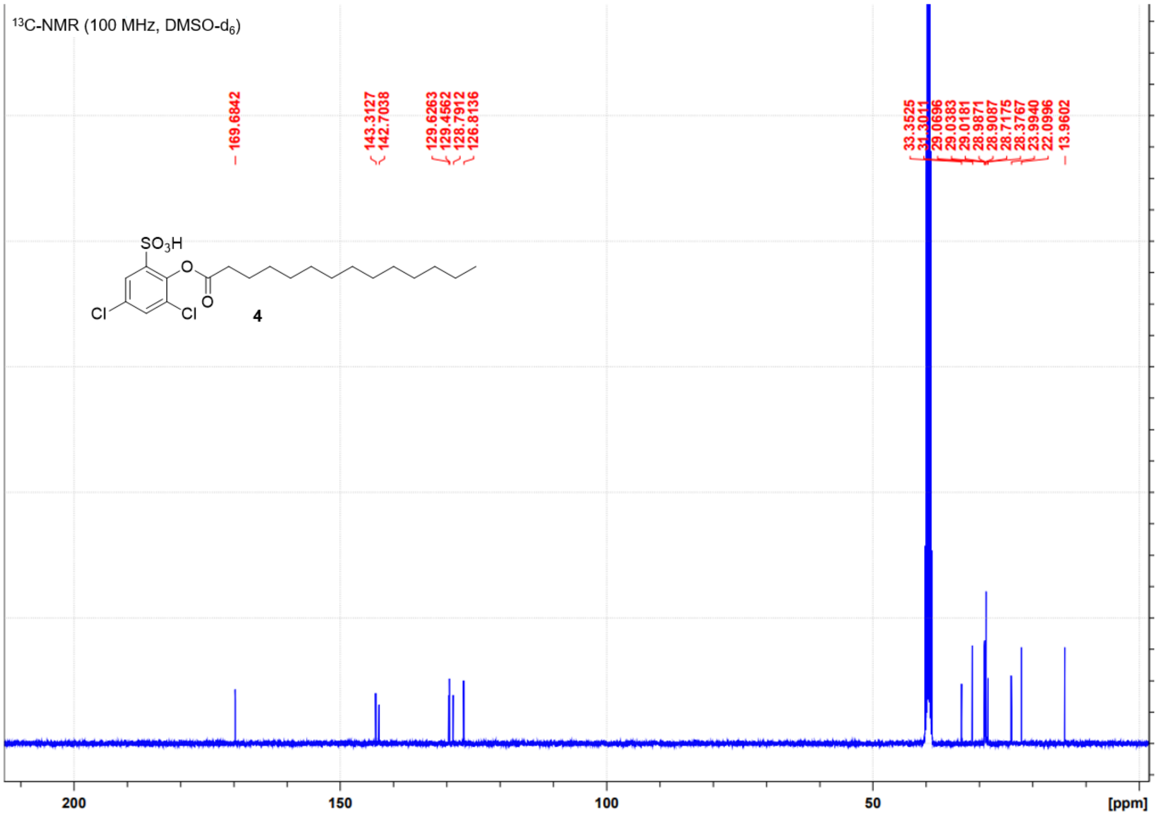

13C-NMR (100 MHz, DMSO-d6)
169.6842
143.3127
142.7038
129.6263
129.4562
128.7912
126.8136
33.3525
31.3011
29.0696
29.0383
29.0181
28.9871
28.9087
28.7175
28.3767
23.9940
22.0996
13.9602
SO3H
O
O
Cl
Cl
4
200
150
100
50
[ppm]
